# Capability of the TFM Approach to Predict Fluidization of Cohesive Powders

**DOI:** 10.1021/acs.iecr.1c04786

**Published:** 2022-02-16

**Authors:** Maryam Askarishahi, Mohammad-Sadegh Salehi, Stefan Radl

**Affiliations:** † Research Center Pharmaceutical Engineering GmbH, Inffeldgasse 13/III, 8010 Graz, Austria; ‡ Institute of Process and Particle Engineering, Graz University of Technology, Inffeldgasse 13/III, 8010 Graz, Austria

## Abstract

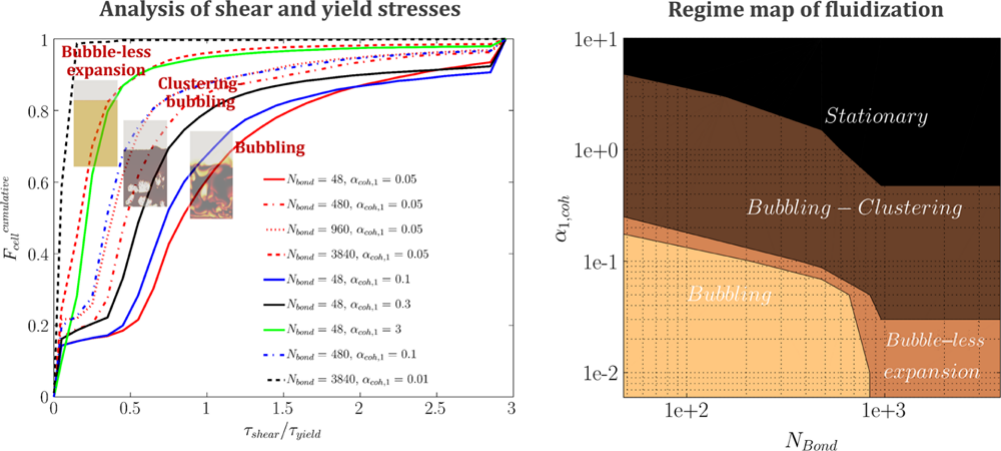

The fluidization behavior of cohesive particles was investigated using an Euler–Euler approach. To do so, a two-fluid model (TFM) platform was developed to account for the cohesivity of particles. Specifically, the kinetic theory of granular flow (KTGF) was modified based on the solid rheology developed by Gu et al. *J. Fluid Mech.***2019**. The results of our simulations demonstrated that the modified TFM approach can successfully predict the formation of particle agglomerates and clusters in the fluidized bed, induced by the negative (tensile-dominant) pressure. The formation of such granules and clusters highly depended on the particle Bond number and the tensile pressure prefactor. To evaluate fluidization regimes, a set of simulations was conducted for a wide range of particle cohesivity (e.g., Bond number and tensile pressure prefactor) at two different fluidization numbers of 2 and 5. Our simulation results reveal the formation of four different regimes of fluidization for cohesive particles: (i) bubbling, (ii) bubbling–clustering, (iii) bubble-less fluidization, and (iv) stagnant bed. Comprehensive analysis of the shear-to-yield ratio reveals that the observed regime map is attributed to the competition between the shear stress and yield stress acting on the particles. The obtained regime map can be extended to incorporate the effect of dimensionless velocity and dimensionless diameter as a comprehensive fluidization chart for cohesive particles. Such fluidization charts can facilitate the design of fluidized beds by predicting the conditions under which the formation of particle agglomeration and clustering is likely in fluidized beds.

## 1 Introduction

### 1.1 Importance of Cohesive Particles in Industry

Small particles are of high interest for various industrial sectors because they offer high volumetric specific surface area, leading to higher contact/reaction rates per unit reactor/bed volume.^2^ For instance, small particles serve as reacting particles in the chemical and petrochemical industry (e.g., pulverized coal combustion and gasification) and are used as solid catalysts for chemical reactions (e.g., fluid catalytic cracking or FCC).^3^ In the pharmaceutical industry, typically, active pharmaceutical ingredients (APIs) are relatively fine to improve the dissolution and release rate in the body. Besides, excipients in the form of fine powders can improve the uniformity of the tablet.^4^ Fine powders are also favorable in the food industry due to their higher solubility.^5^ Fluidized bed technology can be employed to take advantage of such a high specific surface area. However, handling fine/small powders in a fluidized bed (FB) can face several challenges.

Most importantly, small and specifically fine powders categorized as group A and C in Geldart’s classification^6^ are not easy to get fluidized due to the dominance of van der Waals force at small diameters. Powders featuring cohesive interaction forces can be categorized from mildly cohesive to highly cohesive. To quantify the level of cohesion, the Bond number (*N*_Bond_) is typically used. This dimensionless number describes the ratio of cohesion forces and the gravitational force.

Before reaching the bubbling condition, mildly cohesive powder (Group A) typically experiences bubble-less expansion at which the bed gets fluidized uniformly, but no bubble is observed in the bed.^7,8^ Such a behavior can be related to the dominance of yield stress over shearing the powder, which has been the topic of many research studies.^7−11^

A group of researchers evaluated the fluidization behavior of cohesive particles experimentally. For instance, Geldart,^8^ as the pioneer in this field, reported that the interplay of hydrodynamic and interparticle force governs the FB characteristic, for example, bed expansion and minimum bubbling/minimum fluidization ratio.

LaMarche et al.^7^ experimentally investigated the fluidization behavior of mildly cohesive particles for a range of fluidization velocities. Their experimental data suggest that the mean bed voidage is smaller at the minimum bubbling condition due to the dominance of the drag force over the cohesion force. In another study, Li et al.^11^ experimentally investigated the fluidization behavior of Geldart A particles in a fluidized bed. According to their observation, particle cohesion results in the stickiness of a layer of particles on the wall. They also observed the formation of particle clusters in the form of agglomerates which are not permanent as they continuously form and break.

Such experimental observations for cohesive powder fluidization can improve our understanding of particle flow behavior, specifically fluidization regimes. However, experimental studies seem very challenging to obtain a deep insight into the main reason behind such a behavior. This is since one cannot evaluate the contribution of different interparticle forces through experimental investigations. For instance, it is known that the cohesiveness of a powder and its fine content are known to play a significant role in the rheological and fluidization behavior of Geldart’s group A powders.^12^ Specifically, van der Waals cohesive forces can create agglomerates of small particles in a fluidized bed when dominating the applied shear stress.^3^ Here, one can take advantage of the recently established computational models, which we will summarize next.

### 1.2 Mathematical and Computational Modeling

In the study of cohesive powder fluidization, isolating the effects of cohesive (van der Waals) forces from other effects, for example, electrostatics and humidity, is very difficult in experimental investigations.^3^ Capable of predicting spatially distributed information, mathematical approaches enable us to evaluate the effect of individual forces on the flow behavior of cohesive powders. To put it in more detail, detailed numerical simulations are capable of predicting the distribution of interparticle forces and their contributions to the strength of the particle aggregates. Consequently, the flow behavior of such powders can be thoroughly analyzed. Various mathematical approaches have been used in the literature as detailed below.

A group of researchers employed coupled computational fluid dynamics (CFD) and −discrete element method (DEM) simulations to predict fluid (i.e., gas) flow and particle flow, respectively. In this approach, the Navier–Stokes equation is solved for fluid motion, while the motion of individual particles is simulated by solving the second Newton’s law of motion. For instance, Li et al.^11^ used the CFD–DEM approach to evaluate the formation of particle agglomerate in a fluidized bed of Geldart A particles. They reported that DEM simulations fail to capture agglomerates raining down from the bubbles’ cap without accounting for the cohesive forces. Their CFD–DEM results revealed that highly cohesive particles tend to form large agglomerates in dense regions. This finding was also reported by Wu et al.^13^ Their results^11^ demonstrated that the formed agglomerates can be broken on the bubble’s cap. They validated the formation of agglomerates through an experimental study. However, they reported that agglomerates predicted in simulations are larger while having a smaller aspect ratio compared to the experimental observation. They concluded that considering only van der Waals forces in DEM is insufficient to predict the agglomerates’ properties accurately. They related the formation of elongated agglomerates to gas–particle interactions in the presence of cohesive forces, which requires direct numerical simulation. This means that revisiting the fluid–particle interaction is also needed when simulating a fluidized bed of cohesive particles.

In another CFD–DEM study, Liu et al.^14^ reported that the defluidization curve of Geldart A particles depends on the static bed height and Young’s modulus. Their main purpose was to evaluate if the defluidization curve of cohesive powders can be predicted by simulating soft particles (i.e., those featuring low values of Young’s modulus) with the DEM. This was mainly done to reduce the computational cost of a DEM-based simulation. According to Liu et al.,^14^ variation of Young’s modulus can enhance cohesive effects in DEM-based simulations simply because the particle–particle overlap is different (which is an input to the cohesion model). They also examined the effect of the static bed height to obtain a system size-independent measurement for cohesive powders. They attributed this behavior to the interaction of cohesive forces with the packing structure during defluidization and material stiffness. Fluidization/defluidization behavior of aeratable Geldart’s group A powders was also investigated by Galvin and Benyahia.^3^ Their DEM simulation results demonstrated a significant effect of sliding friction in the presence of cohesion. Specifically, they reported that neglecting the effects of cohesion marginalizes the action of friction for small type A particles. Recently, Wu et al.^13^ used the CFD–DEM method to connect the microscopic discrete properties of cohesive powder to the macroscopic continuum description of a cohesive fluidized bed. Their simulation results showed that the tensile pressure predicted by CFD–DEM is in accordance with the Rumpf correlation.^15^

Another group of researchers tried to use microscale simulation (e.g., DEM) to develop closures for a continuum approach (e.g., the two-fluid model, TFM) to simulate cohesive particle fluidization. In the TFM approach, fluid and particles are treated as interpenetrating continua, and the momentum balance equations for the fluid and solids phase are solved. As solid particles are considered as one phase, the computational cost for such an approach is much lower when compared to the CFD–DEM approach, especially for large-scale applications. Therefore, academia and industry have a high interest in equipping the TFM approach with suitable cohesion models.

In the TFM, different approaches have been adopted to account for the effect of cohesion force. The first approach is to directly model the particle growth through, for instance, the population balance equation as suggested by Kellogg et al.^16^ They developed a continuum approach for rapid cohesive-particle flow using such a population balance method. They derived a closure for the success factor of cohesive powders’ collision. Their closure relates the success factors of collision to the granular temperature, a measure of the particles’ impact velocity. They also derived a closure for the effective restitution coefficient, which accounts for the dissipation of energy due to cohesion. Their model contains several model parameters related to agglomeration’s critical velocities, which can be defined using simple discrete element method simulation.

In the second approach, cohesive forces were accounted for in a balance equation of force acting on a single solid particle, as proposed by van Wachem.^17^ Based on this approach, he locally calculated the cohesive agglomerate size, which can be linked to the TFM simulation through solids phases of different diameters, representing primary particles and granules of different sizes. Therefore, the number of solids phases is defined based on the specified bin size in the particle size distribution (PSD).

In the third approach, modified excess compressibility is used to account for the effects of cohesion between particles. Ye^18^ investigated the cohesion effect using discrete particle simulations. Subsequently, they modified the classical KTGF theory according to their soft-sphere discrete parcel method (DPM) simulation results. In detail, they modified the KTGF considering the modified excess compressibility, which accounts for the effects of cohesion between particles. This excess compressibility depends on the magnitude of the cohesive force and the solids volume fraction.

In the fourth approach, as the most robust approach, the rheology of the solids phase was directly modified to account for cohesion forces, as adopted first by Gidaspow and Huilin.^19^ They included a cohesion pressure in their solid pressure model via the radial distribution function used in their KTGF formulation. Very recently, Gu et al.^1^ used the CFD–DEM approach to derive a continuum model for the rheology of mildly cohesive powders. Specifically, they also modified the KTGF theory to account for particle cohesion. Through a CFD–DEM simulation, they modified the constitutive equations for the *solids pressure, solids bulk viscosity and shear viscosity*, and the *rate of dissipation of pseudo-thermal energy*.

### 1.3 Gaps in the Literature

From the abovementioned literature, one can conclude that fluidization/defluidization behavior of Geldart A group particles (i.e., mildly cohesive powder) is not well-understood due to several limitations:

- Experimental evaluation of forces seems very difficult as the effect of force acting on the particle cannot be isolated. Therefore, it seems impossible to explain the reason behind the bubble-less expansion in an FB of Geldart A particles. Mathematical and computational approaches can be a promising option to tackle this issue.
- Lagrangian approaches cannot be applied for large-scale FBs due to the limitation imposed by computational time. Alternatively, less computationally expensive tools such as TFM can be used.
- The standard KTGF fails to predict agglomerate formation in an FB of cohesive particles as this approach has been developed for slightly inelastic particles and does not account for the cohesion force. However, the collisional behavior of particles is influenced by interparticle forces.
- Some researchers coupled the TFM approach with the population balance method to account for agglomerate growth. Nonetheless, the population balance method is associated with several model parameters which are valid for the conditions of establishment only. This limits the applicability of this method. This means that the TFM approach needs to be equipped with robust rheological models for the solids phase to account for the particle cohesiveness, as recently conducted by Gu et al.^1^
- The effect of the Bond number on the fluidization behavior of cohesive powders is not thoroughly investigated in the open literature.
- A detailed regime map of fluidization for Geldart A particles is lacked in the literature. Depending on the level of cohesion, one can expect different regimes of fluidization for mildly cohesive powder: (i) bubble-less expansion, (ii) bubbling, (iii) bubbling–clustering, and (iv) static bed.

### 1.4 Goal and Outline

By considering the gaps in the literature addressed in the previous section, we distilled the following main goals of our present study:

- Evaluating the capability of the modified TFM approach developed by Gu et al.^1^ in predicting (i) the fluidization behavior of cohesive powders and (ii) the formation and breakage of granules. To realize this goal, Gu’s rheological model^1^ was implemented in a TFM simulation platform. Subsequently, the fluidization behavior of cohesive powder was evaluated for different levels of cohesivity in terms of particle Bond number and tensile pressure prefactor (α_coh,1_) in Gu’s model.^1^
- Investigating the influence of particle cohesivity on the solids’ flow properties in a fluidized bed. Specifically, this is the mean solid volume fraction in the emulsion phase and the bed, solid velocity, and the solids’ tensile and compression pressure to quantify the solids’ ability to flow.
- Analyzing the contribution of shear stress and yield stress in the flow properties of cohesive powders for a wide range of *N*_Bond_ and α_coh,1_. To do so, we will evaluate the cumulative distribution of the ratio of shear and yield stress. This analysis enables us to explain the formation of different fluidization regimes for cohesive powders.
- Mapping the granular flow regime for cohesive powders. To achieve this goal, a set of simulations is conducted on a wide range of particle cohesivity (characterized by the Bond number and tensile pressure prefactor) at various fluidization velocities. The obtained regime map will be connected to the cumulative distribution of the shear-to-tensile stress ratio.
- Evaluating how the mixing quality depends on particle cohesiveness. To do so, the mean granular temperature and mean solids velocity variance will be computed for a studied range of cohesiveness levels. These two quantities can be associated with the level of mixing on the particle level (micromixing) and the global level (macromixing), respectively.

To achieve these goals, the open-source code MFiX (Multiphase Flow with Interphase eXchange)^20^ simulation platform was extended to consider the solids rheology developed by Gu et al.^1^ (see Section 2 for the detailed description). In Section 2.1, the full description of the governing equations and the constitutive laws for the kinetic theory of granular flow will be presented. In Section 2.2, the modified solids rheology developed by Gu et al.^1^ will be described. The results of our simulation will be presented in Section 3. Specifically, the performance of an FB of cohesive powders will be analyzed qualitatively and quantitatively in Sections 3.1 and 3.2, respectively. In Section 3.3, the predicted regime map of fluidization will be presented and analyzed based on the ratio of shear stress and yield stress. Finally, we will evaluate the quality of mixing in FBs of cohesive powders in Section 3.4.

## 2 Mathematical Methodology

### 2.1 Hydrodynamics

As described in Section 1.4, the main goal of this study is to evaluate the capability of the TFM approach in predicting the fluidization behavior of cohesive powder. Therefore, the Eulerian–Eulerian approach was applied to simulate the gas–solid flow in the present study. In this approach, different phases are treated mathematically as interpenetrating continua. Conservation equations were derived for each phase and are linked by correlations for interphase transfer rates of momentum. To calculate solid-phase rheological properties, we employed the kinetic theory of granular flow (KTGF) in connection with a frictional stress model.^20^ The complete lists of the governing equations and the constitutive laws have been summarized in Tables 1–3. The cohesiveness of the particles was considered through the modified KTGF model proposed by Gu et al.,^1^ as described in Section 2.2.

**Table 1:** Momentum Equation Used in TFM Simulation

momentum conservation equations for gas and solids phases
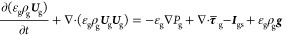 T1.1
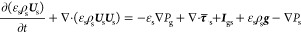 T1.2
interphase momentum transfer
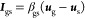 T1.3
momentum exchange coefficient
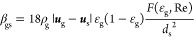 T1.4
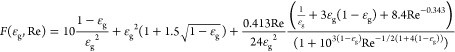 T1.5
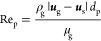 T1.6
solid-phase stress tensor
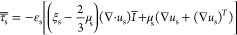 T1.7
solids shear viscosity
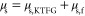 T1.8
solids pressure
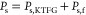 T1.9
gas-phase stress tensor
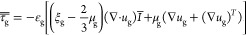 T1.10

**Table 2:** Transport Equation for Granular Kinetic Energy

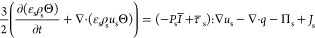 T2.1
Pseudo-thermal conductivity of solids phase
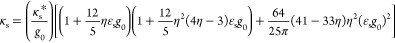 T2.2
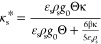 T2.3
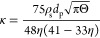 T2.4
Pseudo-thermal energy generation due to the interaction of solids phase with gas
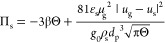 T2.5
Pseudo-thermal energy dissipation due to inelastic particle–particle collision
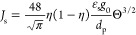 T2.6

**Table 3:** Constitutive Equations for the Calculation of the Solids Stress Tensor

solids viscosity
solids shear viscosity
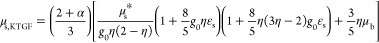 T3.1
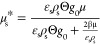 T3.2
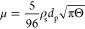 T3.3
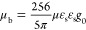 T3.4
solids bulk viscosity
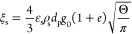 T3.5
solids pressure
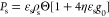 T3.6
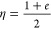 T3.7
frictional stress
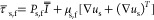 T3.8
solids frictional viscosity
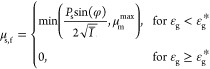
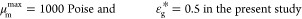 T3.9
second invariant of the deviatoric stress tensor
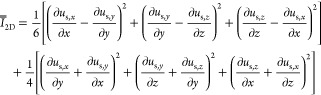 T3.10
frictional solids pressure
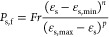
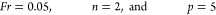 T3.11

### 2.2 Constitutive Models for the Rheology of Cohesive Particles

The rheological model developed by Gu et al.^1^ for cohesive particles (due to the presence of van der Waals forces) was implemented in the MFiX source code. To do so, the conservation equation for granular energy and solid viscosity and pressure were modified, as presented in Table 4. In eq T4.4, *F*_coh_^max^ is the maximum cohesion force, which can be calculated based on the desired Bond number and the particle weight as *F*_coh_^max^ = *N*_Bo_*m*_p_*g*.

**Table 4:** KTGF-Based Models for Cohesive Particles Developed by Gu et al^1^

solids viscosity
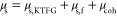 T4.1
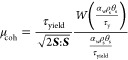 T4.2
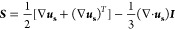 T4.3
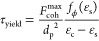 T4.4
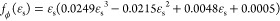 T4.5
solids pressure
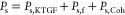 T4.6
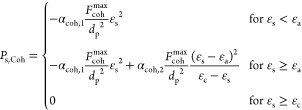 T4.7
Pseudo-thermal energy dissipation (interparticle collisions)
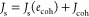 T4.8
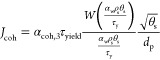 T4.9
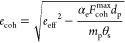 T4.10
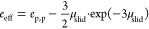 T4.11
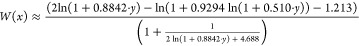
with 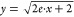 T4.12
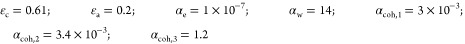

As indicated in eq T4.7, for ε_s_ < ε_c_, the first term (with the prefactor of α_coh,1_) has a negative sign. This means that the force is acting in the direction reverse to the solids pressure. Therefore, one can expect two types of forces between the particles: the first one is the compression pressure which is related to (i) the kinetic pressure associated with the particles’ velocity fluctuations, similar to the pressure in the kinetic theory of gas, and (ii) the force chains formed among the particles that can transmit stress over larger distances.^1^ The latter requires a large particle volume fraction, as indicated in eq T4.7, for ε_s_ ≥ ε_a_ with α_coh,2_ being the relevant prefactor for this term. The second one, which is negative, is the attractive pressure which is attributed to the van der Waals force and acts as a tensile pressure. As the tensile pressure increases, one should expect the formation of larger agglomerates. This means that a higher value of the parameter α_coh,1_ imposes a more significant cohesive force between the particles in the opposite direction in reference to the pressure.

The tensile pressure prefactor, α_coh,1_, represents the attractive pair interaction between the particles. Therefore, it is mainly active in the low solids volume fractions (i.e., ε_s_ ≤ 0.2). Specifically, the tensile pressure prefactor relates the tensile pressure in a granular material to a typical cohesion force between individual particles. According to Gu et al.,^1^ low solids volume fractions do not allow the formation of force chain as the stress cannot be transmitted to a large distance (low coordination number regime). Therefore, attractive van der Waals forces decrease the solids pressure. This force features the sign opposite to the classical compression pressure.

Determining the tensile pressure prefactor is a critical factor in accurately predicting solids pressure. However, this parameter cannot be directly measured, similar to the cohesion parameters in DEM simulations. Hence, a kind of calibration is required. In the original study of Gu et al.,^21^ this parameter has been estimated through DEM-based simulations of homogeneous, simple shear flows of frictional and cohesive particles. They used the Levenberg–Marquardt method^22^ to estimate the value of the tensile pressure prefactor, α_coh,1_. The results of their simulations revealed that α_coh,1_ is a function of the interparticle friction coefficient. Thus, one can relate the tensile pressure prefactor to a typical particle roughness and the tendency of a granular material to show a significant tensile strength.

It should be noted that in the original rheological model of Gu et al.,^1^ the pressure at the volume fraction close to the packed condition of jamming cannot be predicted in a physically meaningful way as the term (ε_s_ – ε_a_)^2^/ε_c_ – ε_s_ will diverge at ε_s_ = ε_c_ and predict an unphysical negative pressure at ε_s_ > ε_c_. The original model of Gu et al.^1^ does not account for the existence of highly packed regions (i.e., ε_s_ > ε_c_). In other words, their study needs to be extended to develop a pressure equation under highly packed conditions. Therefore, using the current version of their model would result in unphysical negative pressures at volume fractions exceeding the jamming point. Therefore, in the present study, the (compressive and tensile) pressure was limited to zero for ε_s_ ≥ ε_c_ to avoid unphysical behavior and convergence problems. More details can be found in Appendix A in the Supporting Information.

In Table 4, *W*(*x*) is the Lambert-W function, which is defined as *W*^–1^(*x*) = *x*·exp(*x*). For numeric purposes, and as suggested by Winitzki,^23^ for *x* > 0, eq T4.12 can be used to approximate this function. As reported by Irani et al.^24^ and Gu et al.,^1^*W*(*x*)/*x* represents the competition between the kinetic energy provided by the shear stress and the cohesive energy.

## 3 Results and Discussion

In the present work, the main focus is given to the capability of the TFM approach in predicting the fluidization behavior of cohesive powers. Therefore, an attempt was made to perform a qualitative and quantitative analysis of solids flow properties. In detail, we first evaluate the capability of TFM in predicting the formation of particle clustering and agglomerate formation qualitatively, as detailed in Section 3.2. The predicted behavior will be analyzed based on the voidage and solids pressure distribution. Subsequently, we will quantitatively examine the effect of particle cohesiveness on solids velocity, solids volume fraction in the emulsion phase, and the bed (see Section 3.3). Afterward, we will propose a regime map of fluidization for cohesive powders by analyzing the distribution of solids pressure, solids shear stress, and yield stress (see Section 3.4). Finally, in the last Section 3.5, the effect of particle cohesiveness on mixing quality will be investigated.

### 3.1 Setup and Parameter Ranges

**Figure 1:**
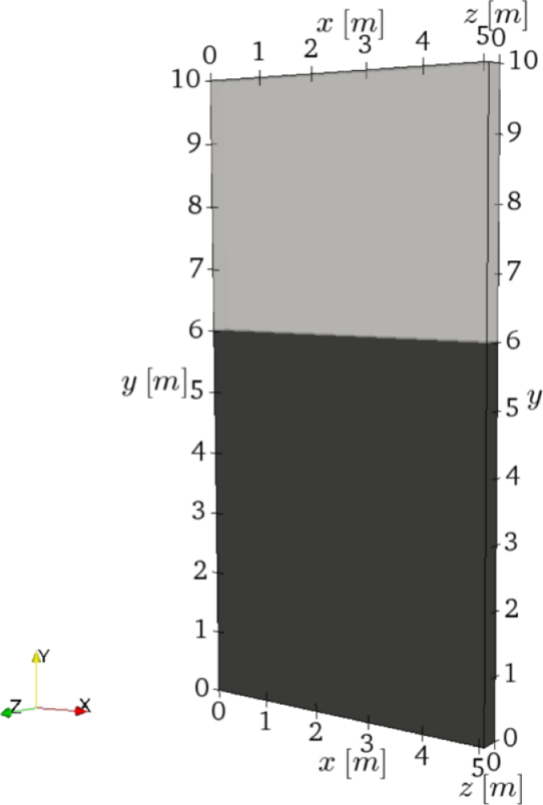
Schematic representation of the studied fluidized bed, the black region shows the dense particle bed at initialization.

To evaluate the capability of TFM in predicting such behaviors, we performed a set of simulations covering a wide range of particle cohesivity at different fluidization velocities. The simulations were performed in a pseudo-2D bed from the study of Li et al.^11^ with a depth of 0.032 m with appropriate wall boundary conditions to model the effect of walls on the fluidization behavior of cohesive powders. A schematic illustration of bed configuration is depicted in Figure 1. Conducting a mesh dependency study revealed that the mesh resolution of 4*d*_p_ is enough to accurately capture particle flow behavior regarding the solids volume fraction and velocity distribution. The Johnson and Jackson boundary condition^25^ was used at the wall for pseudo-thermal granular energy.

The minimum fluidization velocity for noncohesive powders has been defined by performing a set of simulations featuring various fluidization velocities. Subsequently, the resulting bed pressure drop and voidage distribution were analyzed to determine the minimum fluidization velocity. In detail, the pressure drop remains almost constant for *u* ≥ *u*_mf_.

The detailed properties of the particle and fluidization gas and the operating conditions are reported in Table 5.

**Table 5:** Simulation Conditions and Parameters Used in the TFM Approach

parameter	base case	studied range
bed geometry		
*H*_bed_ [m]	0.13	
*L*_bed_ [m]	0.0508	
*W*_bed_ [m]	0.0032	
*H*_0_ [m]	0.06	0.06–0.12
particle properties		
ρs [kg/m^3^]	1500	
d_p_ [μm]	148	
*e*_w,p_ [−]	0.955	
*e*_pp_ [−]	0.9	
*N*_Bo_ [−]	960	0–3840
α_coh,1_ [−]	3 × 10–3	0–10
*u*_mf_ [m/s]	1.73 × 10–2	
gas-phase properties		
ρg [kg/m3]	1.188	
μg [Pa·s]	1.79 × 10–5	
u [m/s]	5*u*mf	2u_mf_–5u_mf_
u_t_ [m/s]	0.649	
Rep (ρg*u*t*d*p/μg)	7.03	
St_p_ (1/18ρp/ρgRep)	451	
wall boundary condition		
gas	no-slip	
solid	partial-slip	
granular temperature	Johnson and Jackson	

### 3.2 Qualitative Analysis of the Particle Cohesion Effect on the Solid Flow Behavior

As mentioned earlier, cohesive powders exhibit different flow behaviors based on the cohesiveness level. Therefore, in this section, we will examine how the particle Bond number can influence the solid particle interactions in the form of particle clustering and agglomeration. We will also evaluate how the particle Bond number can influence the powder flow near the wall due to cohesion.

#### 3.2.1 Qualitative Behavior of the Fluidized Bed of Cohesive Powders

##### 3.2.1.1 Agglomeration Formation

**Figure 2:**
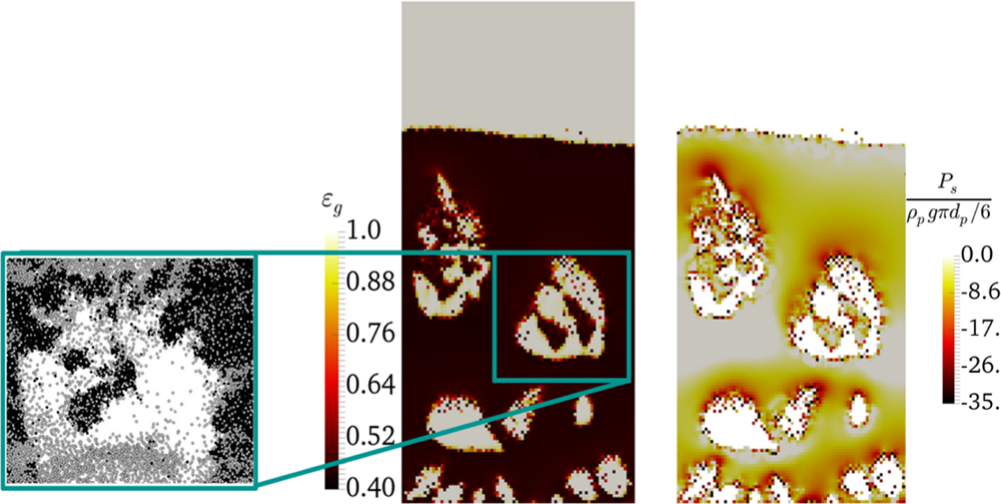
Distribution of negative solid pressure (right panel) and voidage (center panel) upon the formation of agglomerates in the bubbles for *N*_Bond_ = 3840 and α_coh,1_ = 5 × 10^–2^ at *u* = 5u_mf_ and a qualitative comparison with the data of Li et al. (the most left panel).

The main difference in fluidization of noncohesive and cohesive powders is the flowability of the powder and the formation of particle clusters in the bed. Therefore, we conducted a set of simulations for different Bond numbers to see if the agglomeration formation is predicted. The results of simulations revealed that in the studied range of the Bond number, with the model parameters obtained by Gu et al.,^1^ no agglomeration was observed. As described in Section 2.2, the formation of agglomerates can be closely attributed to the presence of tensile (i.e., negative part) solids pressure. This means that the tensile pressure is too low to form agglomerates in the bed. The prefactor α_coh,1_ partially contributes to the tensile pressure between the particles constituting the cohesive powder. Therefore, it seems that the default value of 3 × 10^–3^ represents the behavior of mildly cohesive powder only. Hence, a higher value is required to see agglomerates forming in the bed. Therefore, when we increase the value of the α_coh,1_ parameter, particle clustering and agglomerate formation can be predicted, as shown in Figure 2. This can also be supported by the CFD–DEM results of Wu et al.^13^ They compared the negative solids pressure by CFD–DEM with the one from Gu’s correlation. They reported that a higher value of α_coh,1_ is required to match the tensile pressure. The distribution of negative pressure is presented in the right panel in Figure 2, for the sake of clarity. As shown in this figure, negative pressure can be observed on the bubble caps and partially inside the bubbles.

**Figure 3:**
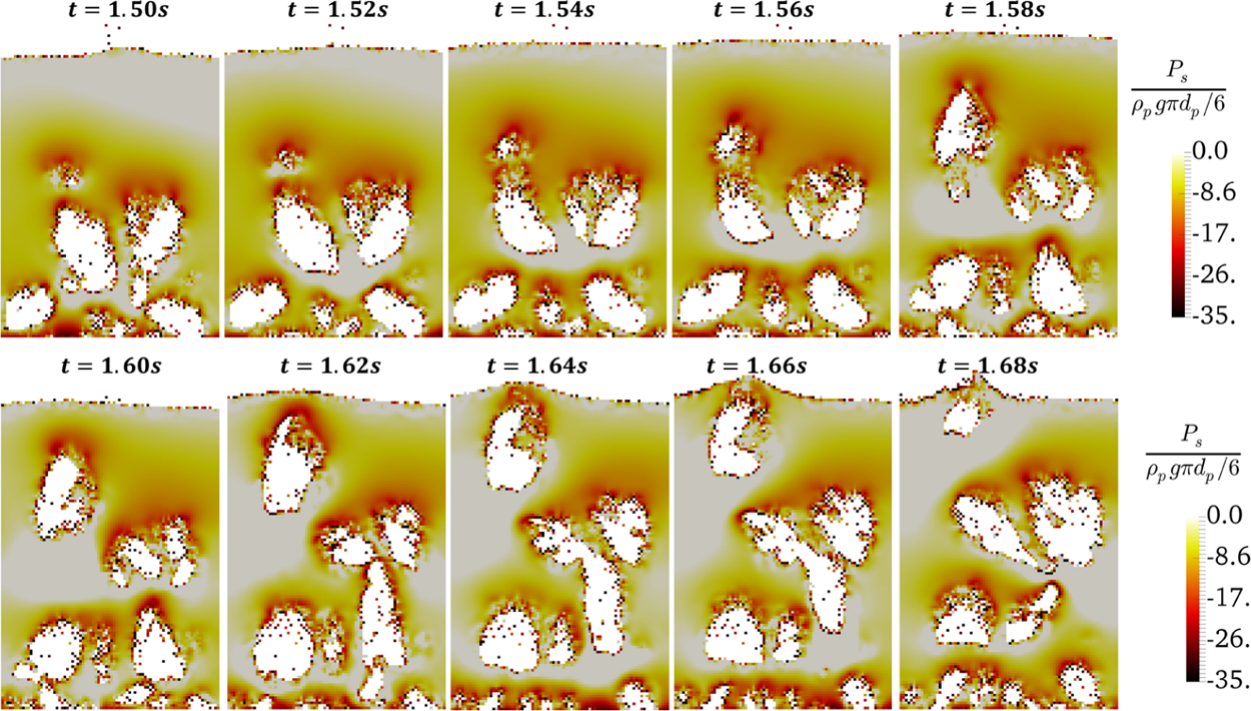
Flow path of the agglomerates (with negative pressure) in the bubble over time for *N*_Bo_ = 3840 and α_coh,1_ = 5 × 10^–2^ at *u* = 5*u*_mf_.

As seen in Figure 2, the agglomerates/clusters are observed on the bubble cap. This predicted behavior is in accordance with the data reported by Li et al.^11^ The high negative pressure is observed on the bubble cap where particle cluster formation can emerge. However, Figure 3 suggests that the highest negative pressure (indicated by the black color) is predicted at the interface of bubbles with the emulsion phase and specifically for the particle traveling back to the bed surface after bubble bursting in the splash zone.

It should be highlighted that Gu’s model does not predict the pressure under packed conditions above the jamming point. The reader is referred to the discussion Section 2.2 for more details. We conducted simulations with an alternative model formulation (i.e., unlimited pressure for ε_s_ ≥ ε_c_), and the results of these simulations are presented in Appendix A of the Supporting Information.

**Figure 4:**
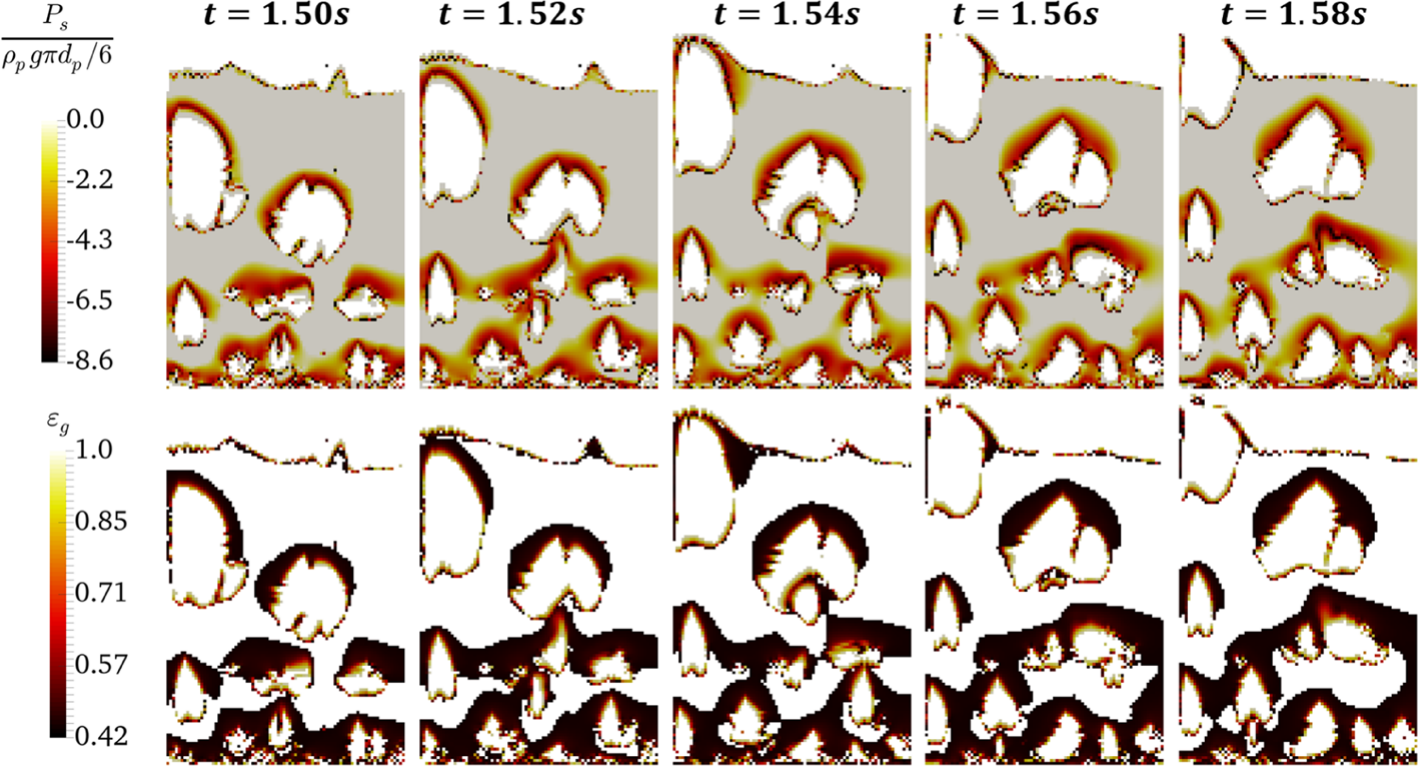
Distribution of voidage in the agglomerate over time for *N*_Bo_ = 960 and α_coh,1_ = 5 × 10^–2^ in a slice located at the center of the bed at *u* = 5*u*_mf_.

**Figure 5:**
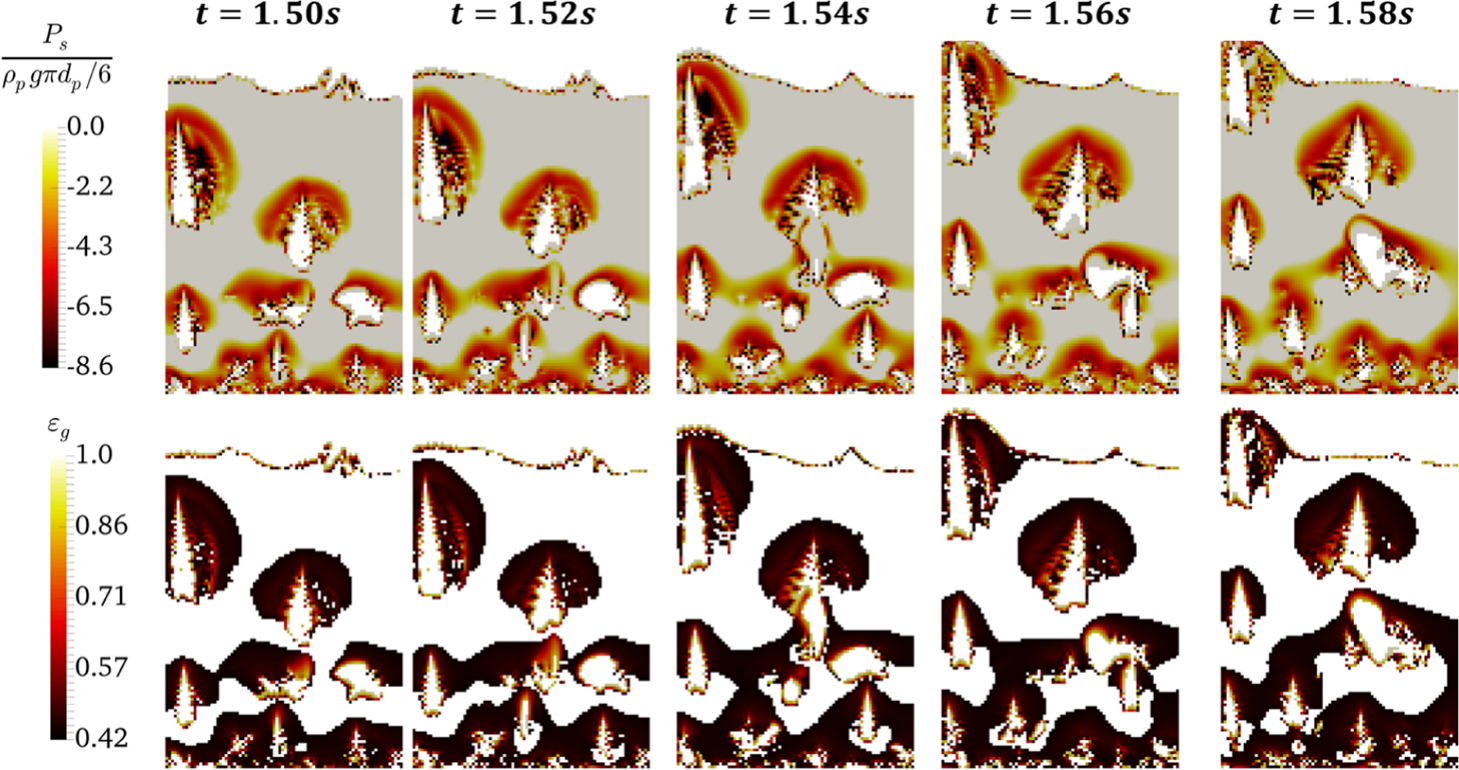
Distribution of negative pressure for the emulsion phase (with ε_s_ ≥ 0.3) (top panel) and the distribution of voidage in the agglomerate (with negative pressure) over the time for *N*_Bo_ = 960 and α_coh,1_ = 5 × 10^–2^ (bottom panel) for the face close to the wall (*z* = 0.001 cm) at *u* = 5*u*_mf_.

The flow path of the agglomerates is shown in Figures 3–5 in more detail. As seen in Figure 3, small particle agglomerates and large clusters can be observed in the bubble and below the bubble cap, respectively (e.g., at *t* = 1.52 s). These clusters agglomerate and form a larger cluster while the bubbles rise in the FB. This breaks the bubble into several smaller bubbles (e.g., see three small bubbles in the right-top half of the bed at *t* = 1.6 s of Figure 3). Subsequently, the clusters are easily disrupted and merged again into the emulsion phase. This leads to the positive pressure in the bubble wake, as seen in Figure 3. On the other hand, the small agglomerates are carried by the bubble, and they can break on the bed surface during bubble bursting, where a relatively high negative pressure is predicted for the solids phase (Figure 5).

The predicted temporary agglomerates can be considered as dynamic aggregates, as suggested by Valverde.^10^ According to Valverde,^10^ agglomerates can be categorized as (i) dynamic aggregates and (ii) cohesive aggregates. Dynamic aggregates can easily break by the gas flow, and their size is limited by the balance between interparticle forces and shear forces. On the other hand, the gas flow cannot disrupt cohesive aggregates.

Nonetheless, these clusters feature relatively weak bonds and can easily break (Figure 4); a clearer visualization of the granule path is provided in a video (Appendix B in the Supporting Information). The agglomerates with higher negative pressure cannot be carried by bubble rise motion and fall into the emulsion phase (see Figure 4, the panels for *t* = 1.56–1.58 s). In Figure 4, the motion of clusters on the bubbles’ caps (denoted by negative pressure in the bottom panel) has been presented for a less cohesive powder (*N*_Bo_ = 960). Upon comparing with the bed of particles with a higher Bond number of 3840 (Figure 3), three points can be noticed:

i. The maximum tensile pressure is lower for less cohesive powders. This results in the formation of a much smaller number of agglomerates in the bubbles for *N*_Bo_ = 960. As seen in Figure 4, no agglomerate is predicted in the bubbles in the top half of the bed. Nonetheless, a small number of agglomerates can be observed close to the distributor surface.
ii. The clusters formed in the bubble are much smaller than the ones in *N*_Bo_ = 3840, which can also be related to the smaller tensile pressure for the lower Bond number.
iii. For both Bond numbers, a relatively high negative pressure is predicted above the bubble’s cap, where particle clusters are more likely to be observed. However, a larger region is influenced by the negative pressure for *N*_Bo_ = 3840 over the bubble’s cap. This behavior suggests that cluster formation is more likely for more cohesive powders, as one would also expect from common sense.

To evaluate the effect of the Bond number on fluidization of cohesive powder, we carefully analyzed different numerical and experimental studies from the literature. Several points can be understood:

i The value of the Bond number highly depends on how the cohesive force is calculated. For instance, in the study of LaMarche et al.,^7^ the van der Waals force is defined based on an asperity radius and the height related to the particle roughness parameter. In their study, for a particle size of 70 μm, a Bond number of 7 and a maximum contact force of 18 nN were reported. However, when using the method of Gu et al.,^1^ the values of 228 and 1004 nN were obtained for this powder. The main reason for such a difference is considering the small-scale roughness parameters in the study of LaMarche et al.^7^ Thus, smooth particles can lead to extremely high Bond numbers.
ii The fluidization behavior of cohesive particles cannot be purely represented by the Bond number. As demonstrated in the present contribution, the solids pressure also plays a key role in the formation of agglomerates and particle clusters. The solids pressure is governed by the Bond number and the tensile pressure prefactor. This can also be supported by comparing the simulation results of Gu et al.^1^ and Wu et al.^13^ In the study of Gu et al.,^1^ the Bond number is in the range of 100–4000 with a tensile pressure prefactor of 3 × 10^–3^. On the other hand, in the study of Wu et al.,^13^ the Bond number is smaller than 20, while α_coh,1_ = 682–785. This means that reducing the Bond number requires a higher tensile pressure prefactor to exhibit similar solids pressure and fluidization behavior in terms of particle agglomeration and cluster formation. This finding has also been reported by Wu et al.^13^
iii The study of Gu et al.^9,21^ shows the dependency of α_coh,1_ on the particle friction coefficient. Therefore, having considered the role of α_coh,1_ alongside the Bond number in fluidization behavior, the formation of agglomerates is indirectly governed by particle friction. This can also be supported by the study of LaMarche et al.,^7^ who included particle friction (through roughness) in the definition of the Bond number (see the discussion in Point i).

##### 3.2.1.2 Particle Flow Behavior near the Wall

It would also be of interest to see how the particle cluster formation and their flow are influenced by walls. To evaluate this behavior, the contour plots for the solids negative (tensile-dominated) pressure and the voidage on the wall have been depicted in Figure 5. Comparing Figures 4 and 5 (shown in a contour plot at the center and the wall, respectively) reveals the formation of larger clusters close to the wall, followed by shrinkage of the bubbles. As seen in Figure 5, a broader region on the wall experiences negative pressures compared to the center of the bed. In addition, the bubbles appear much more elongated close to the wall because of large clusters.

In order to evaluate the effect of the wall on the fluidized bed performance, we performed an additional simulation for the case with *N*_Bond_ = 3840 and α_coh,1_ = 5 × 10^–2^ with a static bed height of 12 cm (i.e., twice the size of the base case). The same fluidization regime as the one in the base case was predicted. Moreover, the results of the performed simulation demonstrated that the solid-phase flow behavior is not affected by the static bed height. Specifically, the solids (bed-averaged) volume fraction changed by 1.09%, the solids mean velocity changed by 4.4%, and the mean granular temperature changed by 6.3%. This finding is also in accordance with the experimental data of LaMarche et al.,^7^ who reported that mean bed voidage is independent of the bed size.

#### 3.2.2 Bond Number Effect on the Flow Behavior

In this section, we aim on quantifying the effect of particle cohesiveness on the solid flow behavior. In detail, we will evaluate the effect of the Bond number on the flow quantities of solids phase, including solids volume fraction, solids compression pressure, and tensile pressure under the steady-state and dynamic conditions as detailed below.

##### 3.2.2.1 Time-Averaged Voidage Distribution

**Figure 6:**
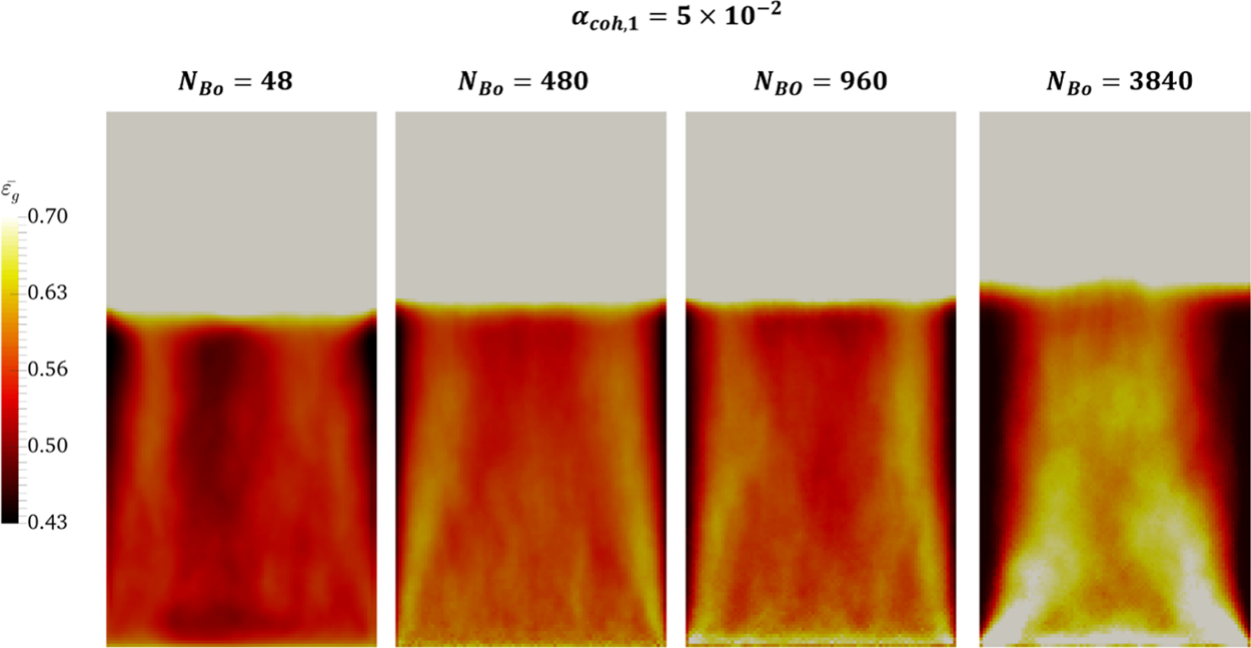
Effect of the Bond number of the time-averaged voidage distribution in the center slice of the fluidized bed at *u* = 5u_mf_.

The contour plot for time-averaged voidage has been depicted in Figure 6. According to this figure, a more uniform distribution of voidage in the bed was predicted for lower Bond numbers. This will also be supported later by the cumulative distribution of the solids volume fraction in the FB, as reported in Section 3.4. This can be attributed to the motion of particles as a cluster in the bubble at high Bond numbers (e.g., *N*_Bo_ = 3840), where bubble coalescence occurs at lower heights relative to the distributor. On the other hand, at low Bond numbers, bubbles prefer to flow in the off-central positions, as indicated by the regions featuring a low voidage in Figure 6. The time-averaged voidage distribution in this figure indicates a higher bed expansion for higher Bond numbers. However, as discerned in Figure 9, the mean voidage in the emulsion phase reveals that a denser emulsion phase was predicted for more cohesive powders (higher *N*_Bond_). This is valid for the cases in which the bed operates in the bubbling or bubbling/clustering regimes. Another point discerned from Figure 6 is that a dense-solid region was predicted near the walls, close to the bed surface (freeboard) for the studied range of Bond numbers. This region is expanded toward the distributor when increasing the Bond number.

##### 3.2.2.2 Time-Averaged Solid Pressure Distribution

**Figure 7:**
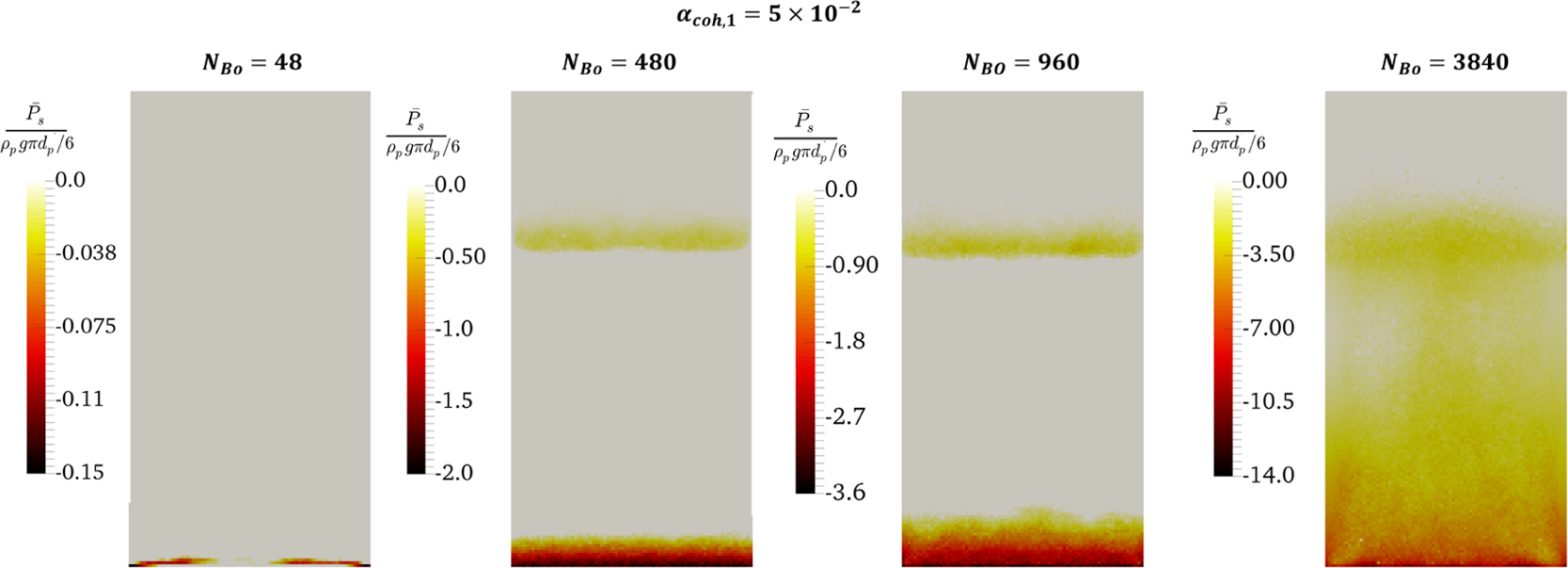
Effect of the Bond number on the time-averaged negative solids pressure (tensile-dominant pressure) in the center slice of the fluidized bed at *u* = 5*u*_mf_.

**Figure 8:**
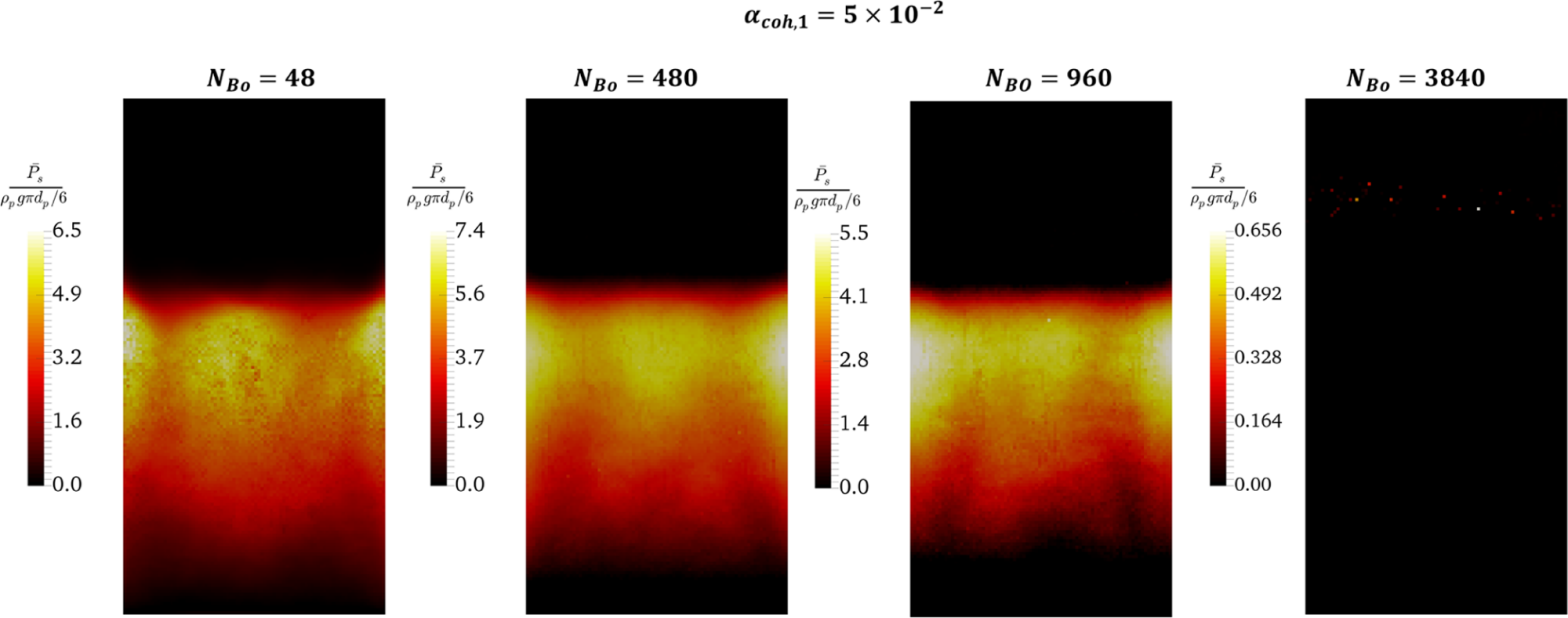
Effect of the Bond number on the time-averaged positive solids pressure (compression-dominated pressure) in the center slice of the fluidized bed at *u* = 5*u*_mf_.

The distribution of the solids’ negative (i.e., tensile-dominant) pressure and positive (i.e., compression-dominant) pressure is shown in Figures 7 and 8. At constant α_coh,1_, a wider region featuring negative pressure is predicted for the higher Bond numbers. In detail, at a very low Bond number (e.g., 48), negative pressure is only observed in the region immediately above the distributor surface (Figure 7, the most left panel). However, at a higher Bond number of 480, negative pressure was also predicted near the bed surface in addition to the distributor. The negative pressure on the bed surface is attributed to the small agglomerates residing on the bed surface (splashing zone), as shown in Figure 5. These agglomerates are carried over by the bubbles to the bed surface, or they may be formed when large agglomerates break on the surface through bubble bursting. Therefore, one can expect a negative time-averaged pressure on the bed surface, which is dominated by tensile pressure. As seen in Figure 7, an increase in the Bond number expands these two regions in the axial direction inside the dense bed. This expansion continues at higher Bond numbers (e.g., 3840) to the extent that negative (tensile-dominated) pressure can be observed everywhere in the dense bed (first right panel of Figure 8 for compression-dominated pressure). It should be noted that as legend bars in Figure 7 indicate, the normalized negative pressure increases with the Bond number.

In Figure 8, the distribution of time-averaged positive (compression-dominated) pressure was presented. As shown in this figure, positive pressure is observed in the top-half region of the bed. The highest compression-dominated pressure is observed immediately below the top negative-pressure region. This is associated with the high solids volume fraction in the top half of the bed (see Figure 6), which results in powder compression. As we approach the distributor surface, the compression-dominated pressure decreases and the tensile pressure becomes dominant.

**Figure 9:**
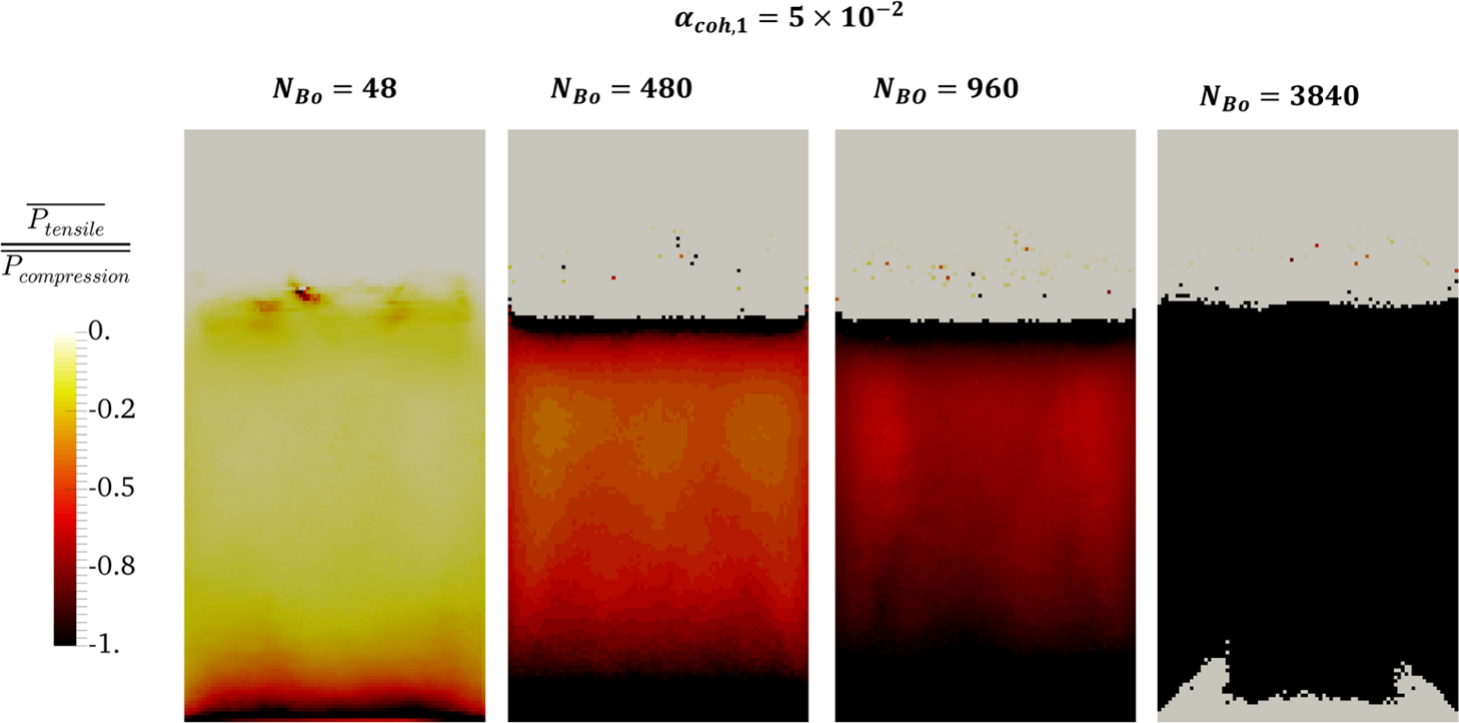
Comparison of the time-averaged ratio of tensile and compression pressure for different Bond numbers in the center slice of a fluidized bed at *u* = 5·u_mf_.

In order to obtain an insight into the contribution of tensile pressure and compression pressure to the total pressure, we computed the ratio of time-averaged tensile pressure and the compressive part based on eq T4.7. As shown in Figure 9, as the particle Bond number increases, a larger area of the bed experiences . As easily discerned from this figure, at *N*_Bond_ = 3840, the tensile pressure is dominant in dense bed regions, except for the one close to the distributor. This means that the probability of agglomerate formation is much higher for this Bond number. The predicted distribution is also in accordance with the distribution of tensile-dominated pressure and compression-dominated pressure shown in Figures 7 and 8, respectively.

### 3.3 Quantitative Analysis of the Particle Cohesion Effect on the Solids Mean Flow Properties

It is also of interest to see how the particle cohesiveness influences the mean solids phase properties in the FB quantitatively. Therefore, the solids flow properties have been quantified through (i) solids volume fraction in the emulsion phase and the bed and (ii) the mean solids velocity in the bed. To achieve this goal, we investigated the effect of the Bond number and α_coh,1_ parameter on mean solids properties (e.g., domain-averaged solid volume fraction in the bed and the emulsion phase and solids velocity).

**Figure 10:**
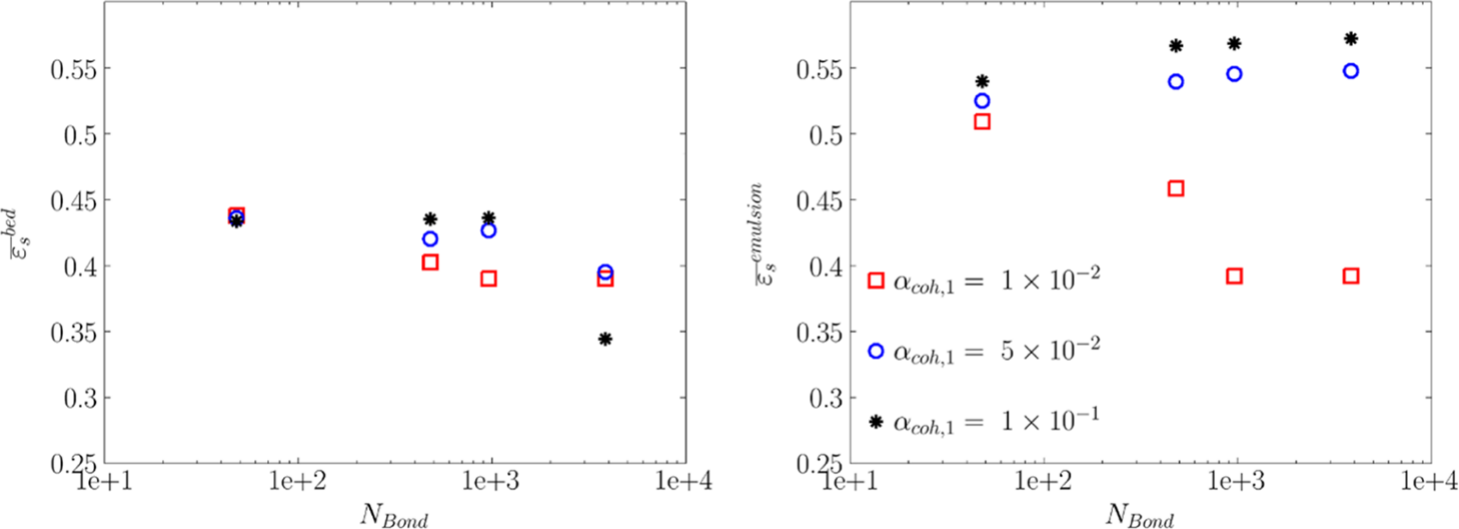
Effect of the Bond number and tensile stress parameter (α_coh,1_) on the time-averaged mean solids volume fraction in the emulsion phase (right panel) and in the bed (left panel) in the studied fluidized bed at *u* = 5*u*_mf_.

We computed the mean time-averaged solids flow properties, such as solids volume fraction in the bed and the emulsion phase (Figure 10), as well as the solids velocity (Figure 15). It was demonstrated that unique trends cannot be extracted for the dependency of these quantities on the Bond number and tensile pressure prefactor. This is since the bed falls into different flow regimes at different values for Bond numbers and the α_coh,1_ parameter (see Section 3.4 for regime mapping).

#### 3.3.1 Solids Volume Fraction Versus Bond Number and α_coh,1_

**Figure 11:**
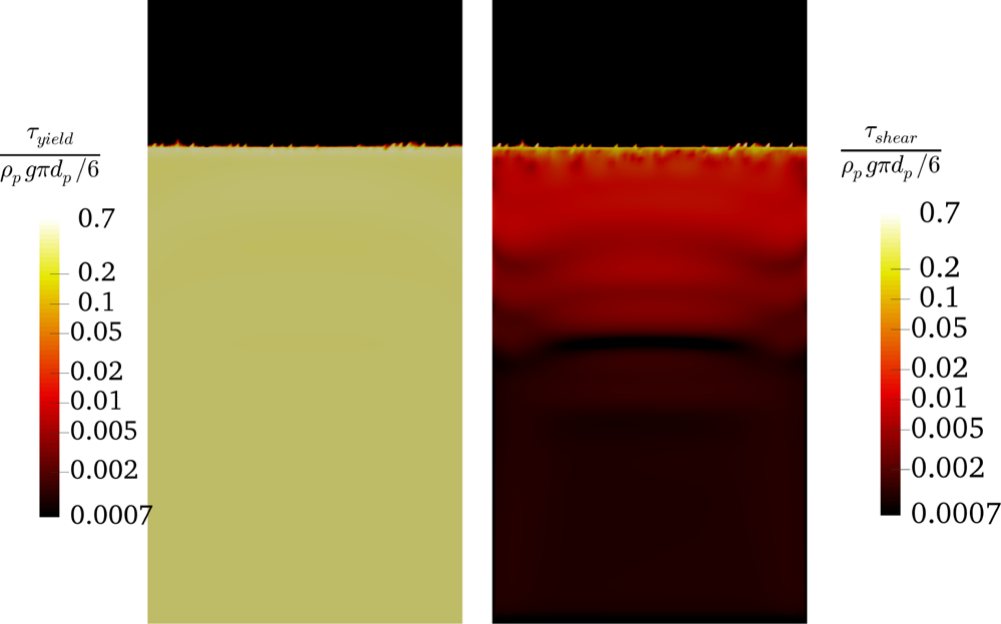
Distribution of solids yield stress and shear stress for the bed experiencing bubble-less expansion (*N*_Bo_ = 3840 and α_coh,1_ = 1 × 10^–2^ at *u* = 5*u*_mf_).

As shown in Figure 10, at a constant Bond number, an increase in α_coh,1_ reduces the solids volume fraction, ε_s_, in the emulsion phase. For α_coh,1_ = 1 × 10^–2^, an increase in the Bond number gives rise to the solids volume fraction of the bed and the emulsion phase. Comparing the right and left panels in Figure 10 revealed that the predicted solids volume fraction is almost similar for the bed and the emulsion phase for *N*_BO_ = 960–3840 and α_coh,1_ = 1 × 10^–2^. The identical voidage of the emulsion phase and the bed indicates that no bubbles are formed, and the bed falls into the bubble-less expansion regime. This can also be supported by the contour plot of the mean voidage shown in Figure 6. The bubble-less expansion of the bed at these two Bond numbers is due to the high yield stress that is larger than the applied shear stress. Figure 11 supports this finding: the yield stress dominates the shear stress approximately by 1 order of magnitude. This means that the fluidization gas cannot shear the particles against the action of cohesion force. As it will be later explained in Figure 15, the solids velocity is zero for these two conditions, demonstrating that the fluidization gas cannot carry particles.

The analysis of bed voidage evolution revealed that the bed initially operates in the bubbling mode (data not shown here). As time passes, the emulsion phase close to the distributor surface rapidly expands (with uniform voidage) and does not allow the formation of bubbles anymore. The height of this expanded bed increases up to the bed surface after a few seconds such that no bubble is observed in the bed, and the whole bed is operated in the bubble-less expansion regime.

However, for higher tensile pressure prefactors (i.e., α_coh,1_ = 5 × 10^–2^ to 10^–1^), a higher solids volume fraction was predicted in the emulsion phase. This reduction in the emulsion phase’ voidage is since the interparticle contacts are disrupted due to bubble passage and solids circulation, as reported by Geldart.^8^ It should be noted that the solids volume fraction in the emulsion phase slightly increases with the Bond number for these two values of the α_coh,1_ parameter.

As the Bond number increases, the effect of α_coh,1_ on the emulsion solids volume fraction is intensified. For instance, a change in α_coh,1_ marginally affects ε_s_ in the emulsion phase at *N*_Bo_ = 48. For α_coh,1_ = 10^–2^, the increase in the Bond number results in higher bed expansion (lower solids volume fraction). An almost similar mean voidage was predicted for Bond numbers of 960 and 3840 due to bubble-less expansion as explained above.

**Figure 12:**
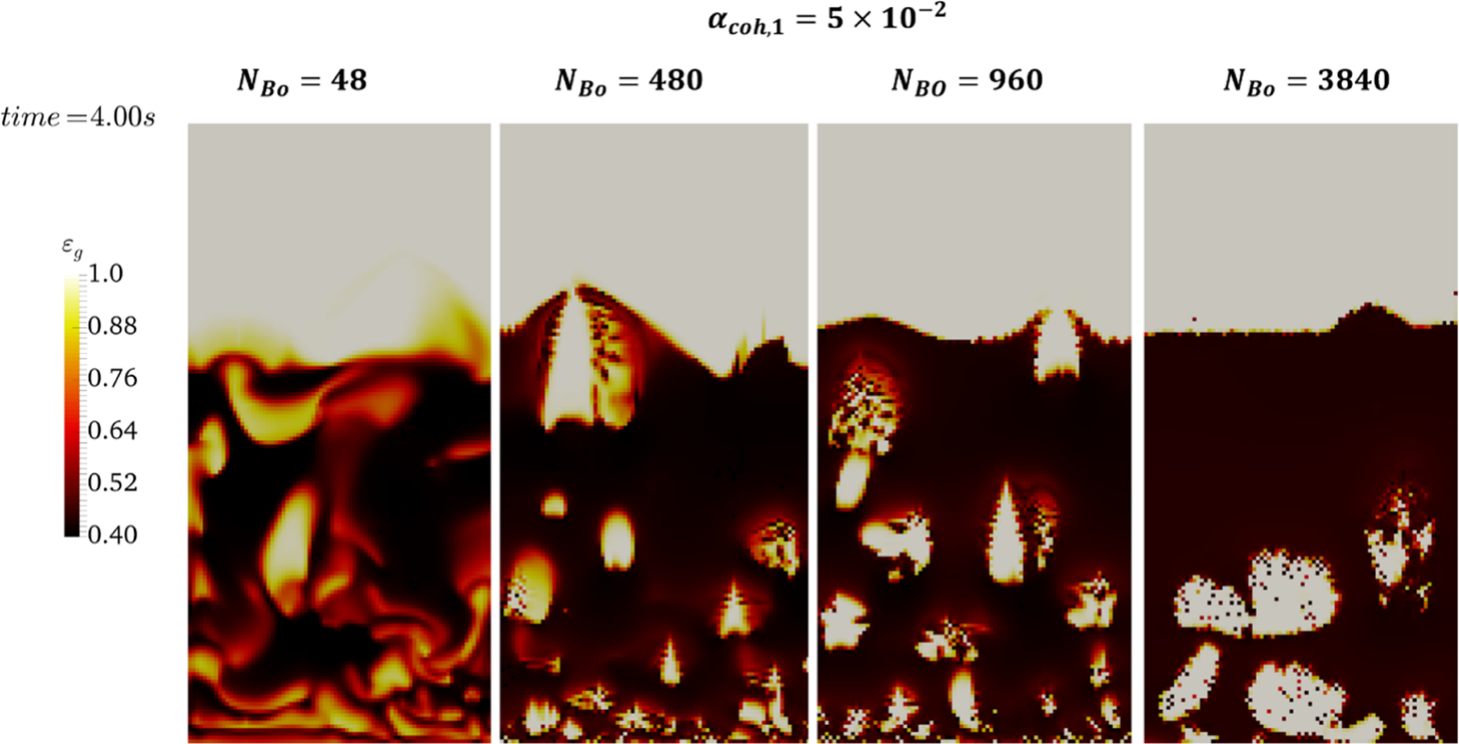
Effect of the Bond number on the instantaneous voidage distribution inside the fluidized bed at *t* = 4 s (α_coh,1_ = 5 × 10^–2^) at *u* = 5*u*_mf_.

**Figure 13:**
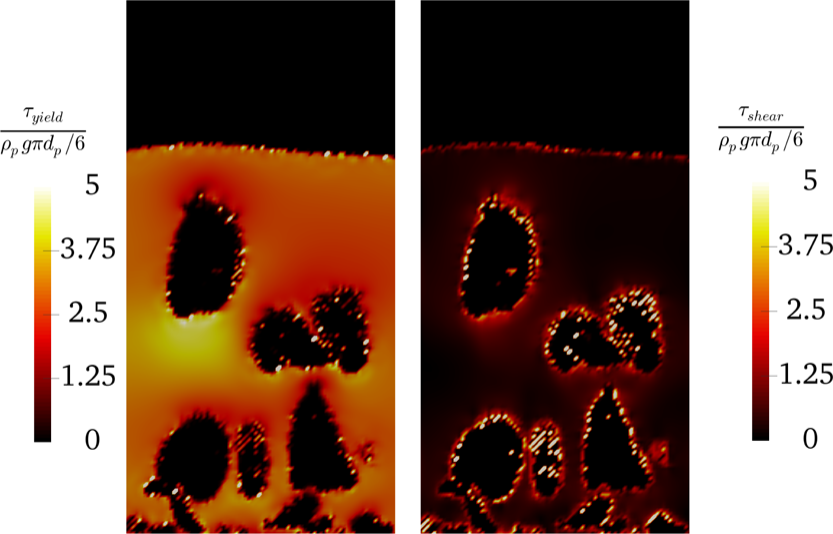
Distribution of the solids yield stress and the shear stress for the bed experiencing particle clustering (*N*_Bo_ = 3840 and α_coh,1_ = 5 × 10^–2^ at *u* = 5*u*_mf_) and a flow time of 1.6 s.

For α_coh,1_ = 5 × 10^–2^, a high difference between ε_s_ in the bed and emulsion phase demonstrated that an increase in the Bond number gives rise to the bubble volumes. This can also be supported by the voidage distribution depicted in Figure 12. This shows that more cohesive powders result in larger bubble formation in the fluidized bed. This behavior can be attributed to particle clustering and agglomeration. The attractive force between the particles is larger for a more cohesive powder. Therefore, the fluidization gas cannot easily shear the particles. This can also be supported by the plot of the shear rate distribution in Figure 13, which demonstrates that the yield stress in the emulsion phase is far larger than the shear stress.

**Figure 14:**
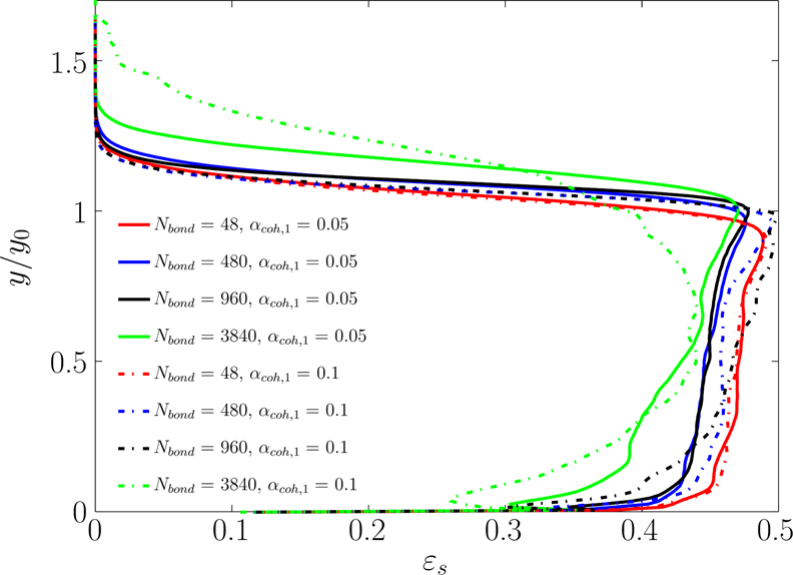
Distribution of the time-averaged solids volume fraction (averaged over the cross-sectional area) along the bed for various Bond numbers and α_coh,1_.

For a detailed investigation, the laterally time-averaged solids volume fraction was computed along the bed. To do so, the time-averaged ε_s_ was averaged over the cross-sectional area at each height. As shown in Figure 14, for *N*_Bo_ = 3840, the level of heterogeneity in ε_s_ is higher compared to that observed for lower Bond numbers. Specifically, for *N*_Bo_ ≤ 960, the voidage distribution is more uniform along the bed excluding the distributor surface and splashing zone. The reason is the formation of large bubbles in the bed filled with the most cohesive powders (*N*_Bo_ = 3840). Such a distribution results in a lower mean solids volume fraction (ε̅_s_^bed^) and consequently higher bed expansion, as supported by Figures 10 and 12. Such a heterogeneity can also be understood by the cumulative distribution of voidage later presented in Figure 17.

#### 3.3.2 Solids Velocity Versus Bond Number and α_coh,1_

**Figure 15:**
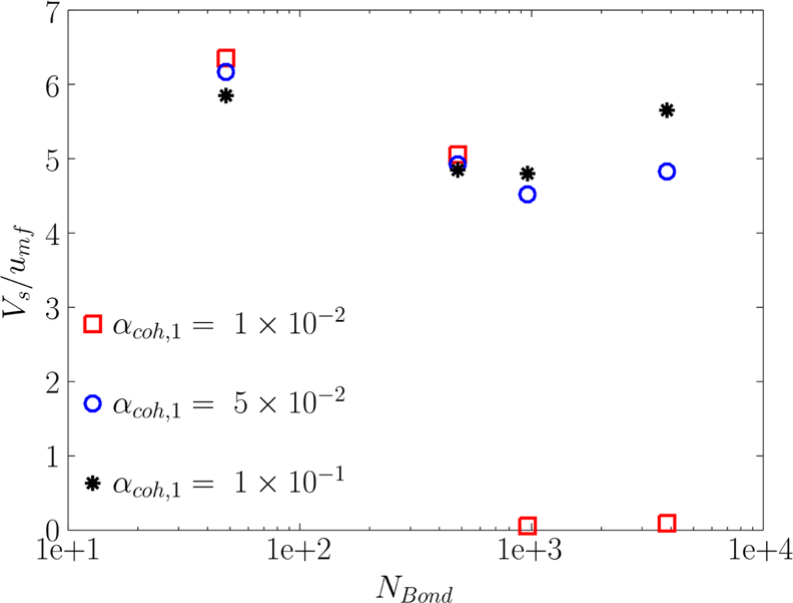
Effect of the Bond number and tensile stress parameter (α_coh,1_) on the time-averaged mean solids velocity in the fluidized bed at *u* = 5*u*_mf_.

The predicted flow behavior can also be explained by considering the solids mean velocity. As shown in Figure 15, the mean solids velocity is more influenced by the Bond number rather than α_coh,1_ in the studied range of particle cohesiveness. The only exception is the cases with *N*_Bo_ ≥ 960 and α_coh,1_ = 1 × 10^–2^. This is since in these cases, the bed falls into the bubble-less expansion regime, and the solids velocity drops to zero. On the other hand, the solids velocity decreases with an increase in the particle Bond number for *N*_BO_ ≤ 960. This can be since the fluidization gas has less capacity to shear the particles due to the increase in the dominance of van der Waals forces with increasing Bond number. This finding highlights that the competition between the shear stress and yield stress plays a significant role in the flow behavior of the FB. As detailed in the following section, such a competition can also influence the flow regime of cohesive powders in the bed.

### 3.4 Predicted Fluidization Regimes

As mentioned earlier, the flow behavior of cohesive powders in a fluidized bed is mainly governed by the competition between the solids shear stress and yield stress. As the yield stress increases, higher shear stress is required to shear the particles and form bubbles in the fluidized bed. In this section, we analyze the ratio of solids shear stress and yield stress. The main objective is to quantify the fluidization regime for cohesive particles through a regime map. To do so, we will first compute the instantaneous voidage and *R*_τ_ = τ_shear_/τ_yield_ for all cells in the simulation region. To do so, the shear stress is computed in each cell as
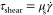
1in which the solids shear viscosity is given by eq T4.2 and the shear rate, γ̇, is given by
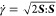
2Here, ***S*** is the magnitude of the rate of the deformation tensor, given by eq T4.3. The expression for the yield stress has been given in eq T4.4. Then, the cumulative distribution of *R*_τ_ was calculated for the studied range of the Bond number and α_coh,1_. Afterward, the predicted regime map will be explained based on the obtained cumulative distribution of *R*_τ_.

#### 3.4.1 Contribution of Shear Stress and Yield Stress

The distribution of *R*_τ_ for a fluidization number, *u*/*u*_mf_, of 5 has been plotted in Figure 15. As shown in this figure, for *a constant value of* α_coh,1_ (e.g., α_coh,1_ = 0.05), an increase in the Bond number leads to a rise in the fraction of cells with dominant yield stress (i.e., τ_shear_/τ_yield_ < 1). This is since the yield stress directly increases with the Bond number, according to eq T4.4.

However, a different behavior was predicted for *a constant Bond number* over the studied range. In detail, for the constant Bond number of 48 and 480, an increase in α_coh,1_ leads to a rise in the fraction of cells with dominant yield stress (τ_shear_/τ_yield_ < 1). On the other hand, the cases with *N*_Bo_ = 3840 and α_coh,1_ = 0.01 deviate from this trend. Therefore, such a trend for the spatial distribution of τ_shear_/τ_yield_ suggests different flow regimes for various combinations of Bond numbers and α_coh,1_.

Specifically, for the case with *N*_Bo_ = 3840 and α_coh,1_ = 0.01, the shear stress is much smaller than the yield stress in all computational cells. This reveals the dominance of the yield stress over shear stress, meaning that the fluidization gas cannot shear the particles and form bubbles. Therefore, bubble-less expansion is expected for such cases.

**Figure 16:**
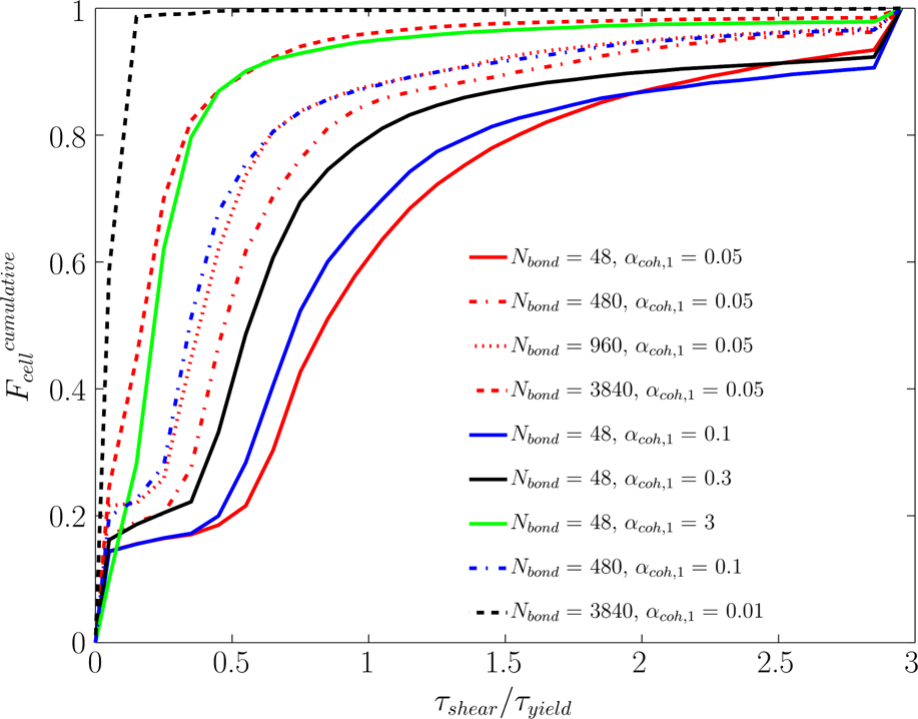
Cumulative distribution of the shear-to-yield stress ratio for various Bond numbers and α_coh,1_ at *u* = 5*u*_mf_.

**Figure 17:**
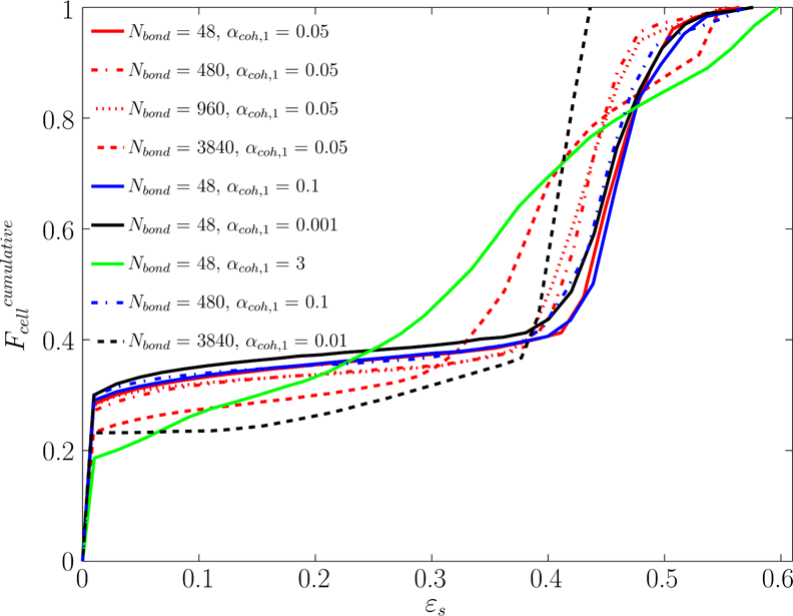
Cumulative distribution of the solid volume fraction for various Bond numbers and α_coh,1_ at *u* = 5*u*_mf_.

To prove this claim, we analyzed the cumulative distribution of the solids volume fraction. As shown in Figure 17, for *N*_Bo_ = 3840 and α_coh,1_ = 0.01, excluding the cell with ε_s_ ≤ 0.05 (as first bin), 80% of cells are occupied by 40–42% solids (ε_s_ = 0.4–0.42). This supports the uniform distribution of solids in the bed. In this manner, in all the cells, the predicted τ_shear_ is smaller than τ_yield_, as seen in Figure 16. This situation surprisingly occurs for high Bond numbers with low α_coh,1_. Such a behavior was also predicted at a lower fluidization velocity of *u*/u_mf_ = 2 and α_coh,1_ = 0 for *N*_Bo_ ≥ 48, while at the lower Bond number of *N*_Bo_ = 4.8 and α_coh,1_ = 0, the bubbling regime was predicted.

Analysis of our simulation results revealed that the smaller the value of α_coh,1_, the lower both the yield stress and shear stress are. However, the shear–yield stress ratio is smaller, demonstrating that the reduction in shear stress is more significant than the yield stress. It should be noted that the solids volume fraction plays a significant role in the yield stress, according to eqs T4.4 and T4.5. Very low values of α_coh,1_ for *N*_Bo_ ≥ 960 resulted in the bubble-less expansion of the bed and consequently lower bed solids volume fraction. This leads to a drop in the yield stress as the yield stress is highly influenced by ε_s_.

Consequently, it sounds not straightforward to explain the predicted behavior by analyzing the shear-to-yield stress ratio due to the interrelation between the voidage, granular temperature, and stresses. It should be added that increasing α_coh,1_ gives rise to the solids negative pressure (tensile-dominant pressure). Therefore, the particle agglomerates are more likely to be formed. Gas can bypass the agglomerates and shear the particles more easily as one can easily see in Appendix C of the Supporting Information. Our simulation results also revealed that an increase in α_coh,1_ gives rise to the solids granular temperature for the cases with dominant shear stress.

#### 3.4.2 Cohesive Powder Flow Regimes in BFB

The analysis of the cumulative distribution of *R*_τ_ = τ_shear_/τ_yield_ reveals that R_τ_ can be considered as a good indicator for the fluidization regimes of cohesive powders. To put in more detail, the predicted cumulative distribution of *R*_τ_ can be categorized into three groups as

i *F*_cell_^cumulative^ (*R*_τ_ < 1) ≅ 1 (e.g., *N*_Bo_ = 3840, α_coh,1_ = 0.01): the bed experiences a bubble-less expansion at the studied fluidization velocity (e.g., 5*u*_mf_). The solids yield stress dominates the shear stress. Therefore, the fluidization gas cannot fluidize the particles, resulting in the uniform distribution of solid particles in the bed. This is also supported by the cumulative distribution of the solids volume fraction shown in Figure 17.
ii *F*_cell_^cumulative^ (*R*_τ_ < 1) ≥ 0.8 (e.g., *N*_Bo_ = 3840, α_coh,1_ = 0.05 or *N*_Bo_ = 48, α_coh,1_ = 3): the bed experiences the formation and breakage of particle clusters. These clusters can be carried by the bubbles and break at the bed surface or flow downward in the bubble and break. Therefore, they are not mostly permanent agglomerates considering the pressure distribution over the bubble cap. As easily discerned from Figure 17, the broader distribution of the solids volume fraction is predicted for this regime, which shows the highest level of heterogeneity in the FB due to the formation of large bubbles.
iii *F*_cell_^cumulative^ (*R*_τ_ < 1) ≤ 0.8 (e.g., *N*_Bo_ = 48, α_coh,1_ = 0.0.5–0.1): the bed is operated in the bubbling regime with the marginal effect of tensile pressure. Therefore, particle clusters are rarely formed. According to Figure 17, the distribution of the solid volume fraction has a higher level of heterogeneity than the bubble-less expansion but is lower than the clustering bed.

**Figure 18:**
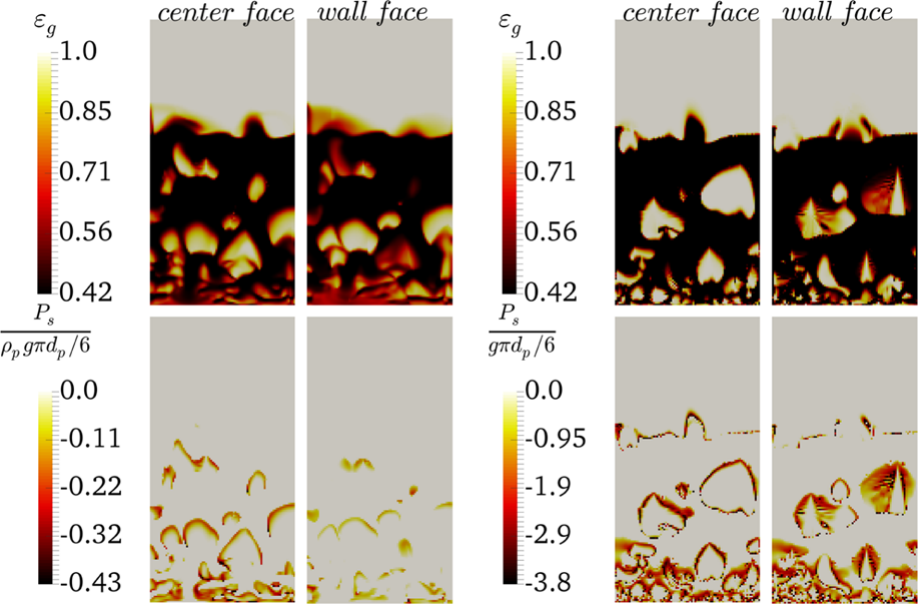
Distribution of voidage and tensile pressure for *N*_Bond_ = 48 and α_coh,1_ = 0.05 (left panel) and α_coh,1_ = 0.3 (right panel) at time = 4 s.

It should be added that a transition zone was identified for 0.5 ≤ *F*_cell_^cumulative^ (*R*_τ_ < 1) ≤ 0.8. In this transition zone, the fluidization regime is mainly bubbling. However, small negative pressure values can be observed near the bubbles’ caps. Nonetheless, the tensile pressure is small compared to the stress caused by the gravitational force. This means that the formation of particle clusters and agglomerates rarely happens in this range. In other words, the higher maximum tensile pressure is expected for the cohesive bed with the higher resultant value of *F*_cell_^cumulative^ (*R*_τ_ < 1). To support this, the distribution of solid voidage and tensile pressure was compared, as shown in Figure 18, for the cases with *F*_cell_^cumulative^ (*R*_τ_ < 1) = 0.5 (*N*_Bond_ = 48, α_coh,1_ = 0.05) and 0.8 (*N*_Bond_ = 48, α_coh,1_ = 0.3). Therefore, the transition zone can be merged to *F*_cell_^cumulative^ (*R*_τ_ < 1) ≤ 0.8.

**Figure 19:**
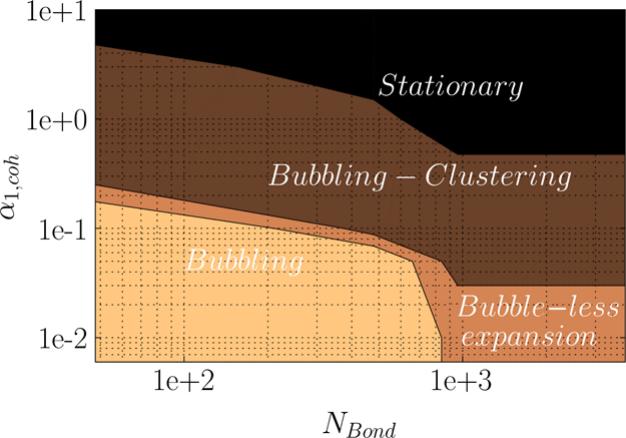
Regime map of fluidization for cohesive powder at *u*/*u*_m_ = 5.

Therefore, four fluidization regimes for cohesive powder can be identified, as shown in Figure 19:

i Shear-dominant (bubbling); at low Bond numbers and low α_coh,1_, particles are easily fluidized with the marginal effect of cohesion. In this regime, the shear stress significantly dominates the yield stress.
ii Yield-dominant (bubble-less expansion): at high Bond numbers and low values of α_coh,1_, the dominance of the van der Waals force does not allow the fluidization gas shearing the particles, resulting in bubble-less expansion of the bed.
iii Intermediate (clustering–bubbling): at intermediate-to-high values of the Bond number and α_coh,1_, the contribution of yield stress and shear stress is comparable. Therefore, particles can form temporary agglomerates which can easily break up with the bubble flow.
iv Purely yield-dominant (stationary): at high values of α_coh,1_, the dominance of tensile pressure results in forming a big agglomerate in the bed. Therefore, the fluidization gas cannot fluidize the particles. It should be added that due to high tensile pressure, the solution can easily diverge.

It should be noted that one of the main purposes of the present work was to evaluate the fluidization regimes at different Bond numbers. Therefore, we have chosen a wide range of α_coh,1_ values (to reflect a wide range of tensile pressures) to observe different fluidization regimes (e.g., bubbling, clustering–bubbling, bubble-less expansion, and stationary) at a fixed Bond number. However, for high Bond numbers (*N*_Bond_ > 960), even at α_coh,1_ = 0, the bubbling regime was not predicted and the bed started to uniformly expand, as one can easily see in the predicted fluidization regime map in Figure 19.

**Figure 20:**
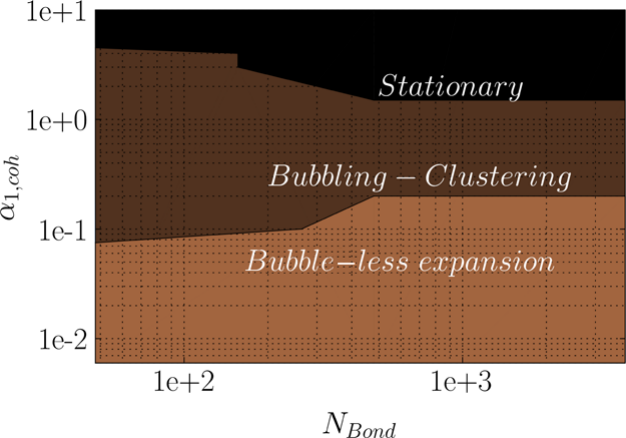
Regime map of fluidization for cohesive powder at *u*/*u*_mf_ = 2.

As the fluidization velocity influences the flow regime in the fluidized bed,^26^ we evaluated the fluidization of cohesive particles at a lower fluidization velocity, *u* = 2*u*_mf_. As easily discerned in Figure 20, compared to the higher fluidization velocity of 5*u*_mf_, the extent of each regime can vary in the design space of *N*_Bond_ – α_coh,1_. Specifically, for *u* = 2*u*_mf_ the bubble-less expansion regime starts at lower Bond numbers (*N*_Bo_ > 48) and lower α_coh,1_ (value of zero). This regime continues up to α_coh,1_ = 5 × 10^–2^ at *u* = 2*u*_mf_ while up to α_coh,1_ = 1 × 10^–2^ at *u* = 5*u*_mf_. The main reason for such a behavior is that at lower fluidization velocity, the shear supported by gas cannot overcome the yield stress between the particles at constant *N*_Bo_ and α_coh,1_. Therefore, the bed cannot get fluidized and falls into the bubble-less expansion. Hence, the bubbling regime is expected to occur at a lower Bond number. To evaluate this, a set of the simulation was performed for a lower Bond number of 4.8; the bubbling regime was predicted for α_coh,1_ ≤ 5 × 10^–2^, which was not shown in the regime map in Figure 20. Therefore, one can conclude that in addition to particle cohesion properties, process parameters of fluidization gas play a significant role in the predicted regime map for cohesive powders.

### 3.5 Mixing Quality

In this section, the effect of particle cohesiveness was evaluated on the mixing quality at the particle level (micromixing) and global level (macromixing). Micromixing is associated with mixing due to the fluctuation of solid particles’ velocity, quantified by granular temperature.^27^ On the other hand, macromixing is attributed to solids mixing due to the motion of bubbles in the fluidized bed, quantified by the solids velocity variance.^27^ More information about the mixing can be found in our previous works.^27−29^

#### 3.5.1 Micromixing (Particle Level)

**Figure 21:**
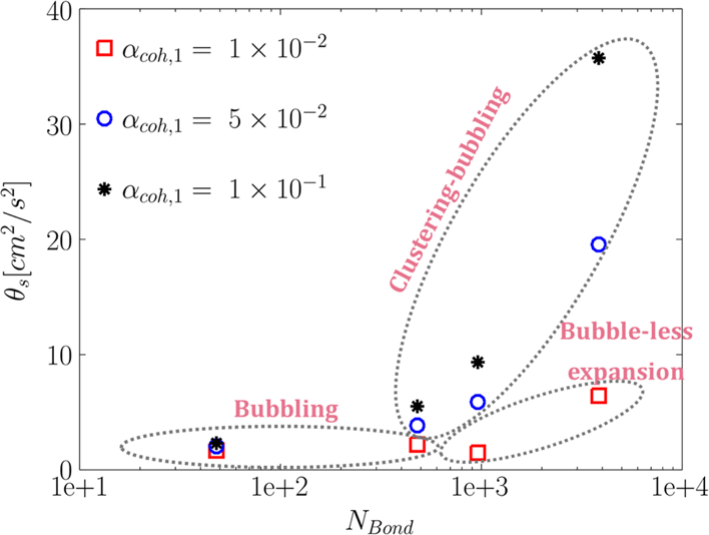
Effect of the Bond number and tensile stress parameter (α_coh,1_) on the time-averaged mean solid granular temperature in the fluidized bed at *u* = 5u_mf_.

**Figure 22:**
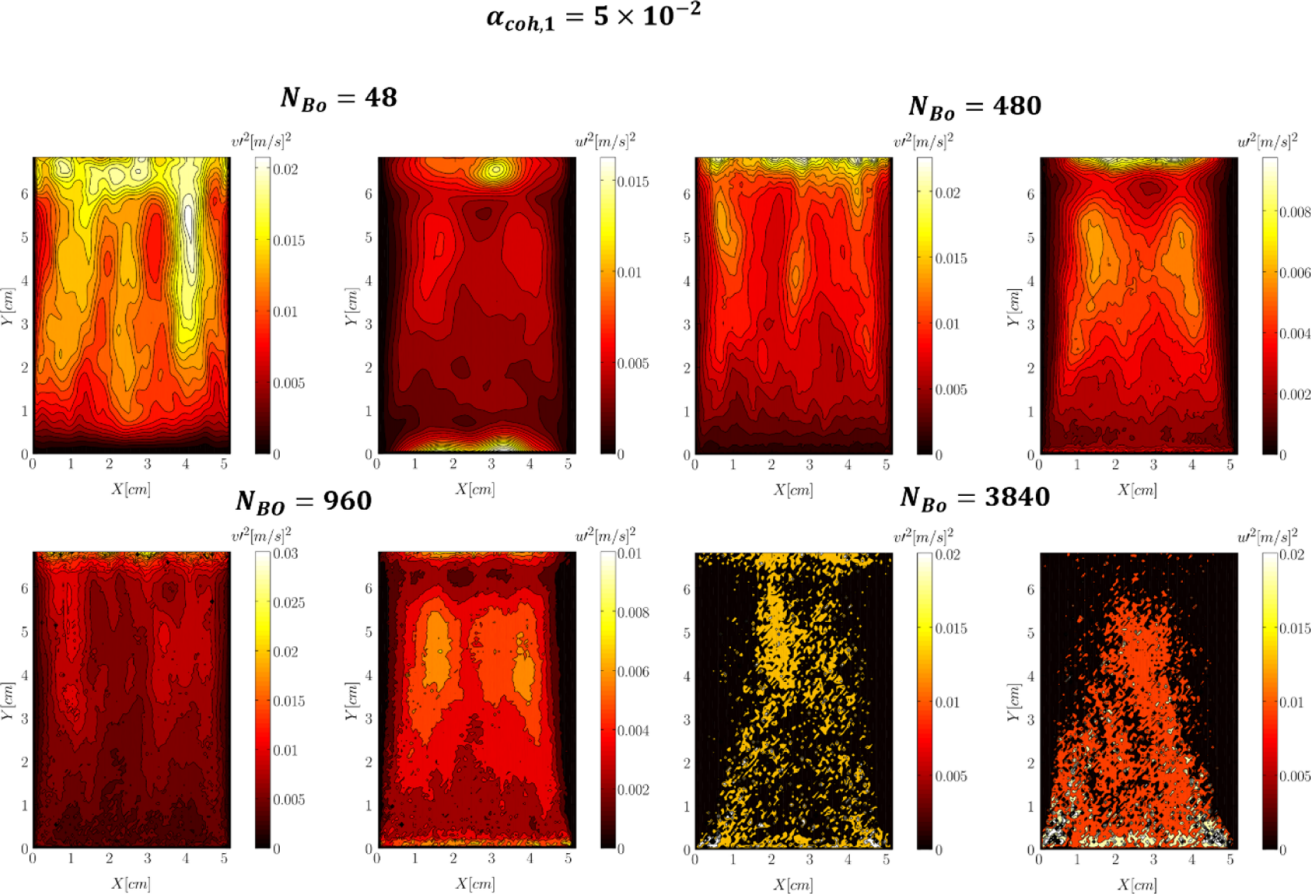
Effect of the Bond number on the solids axial velocity variance (left subpanel for each Bond number) and solids lateral velocity variance (right subpanel for each Bond number) in the fluidized bed for α_coh,1_ at *u* = 5*u*_mf_.

To evaluate the level of micromixing, mean time-averaged solids granular temperature was computed for the studied range of the Bond number and α_coh,1_. To do so, the mean granular temperature was weighted by the solids volume fraction in each cell as
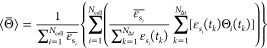
3where *N*_Δ*t*_ is the total number of time steps and *N*_Cell_ stands for the total number of cells in the computational domain. The computed mean granular temperature is depicted in Figure 21. Generally speaking, an increase in the Bond number and α_coh,1_ leads to a rise in the mean granular temperature. The only exception is when the bed falls into the bubble-less expansion regime. This finding is in accordance with our previous study^30^ and the study of Luding^31^ and Radl et al.^32^ This revealed that particle cohesiveness increases the velocity fluctuation at the particle level. However, different behaviors were predicted for macromixing in the bubble level (see Figure 22). According to Figure 21, for the higher Bond numbers, the effect of α_coh,1_ on the micromixing becomes more significant.

One can also analyze the predicted granular temperatures in connection with the predicted fluidization regimes. As discerned from Figure 21, the effect of the Bond number and α_coh,1_ on the particle fluctuation intensity is marginal in the bubbling regime. Such a behavior is expected as the flow of particles is mainly dominated by the shear imposed by fluid flow. However, an increase in the Bond number or tensile pressure prefactor leads to a rise in the granular temperature in the clustering–bubbling regime. Luding^31^ and Radl et al.^32^ also reported this trend. A similar behavior can be observed for the bubble-less expansion regime.

#### 3.5.2 Macromixing (Global Level)

In a fluidized bed, solids macromixing is mainly influenced by the motion of bubbles. Therefore, the variance of the hydrodynamic velocity is of high relevance to quantify macromixing.^33^ In this regard, the solids velocity variance tensor was computed using the following equation in each computational cell^27^
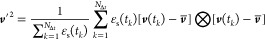
4where ***v*®** and ***v***(*t*_*k*_) represent the time-averaged and instantaneous solids velocity in the computational cell, respectively. ⊗ denotes the dyadic product. Solids mean velocity, ***v*®**, was weighted by the solids volume fraction and is given by
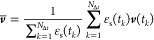
5

In Figure 22, the distribution of velocity variance was depicted for various Bond numbers and a constant tensile pressure prefactor of α_coh,1_ = 5 × 10^–2^. This figure suggested that the lowest velocity variance can be predicted close to the wall. This is because the bubbles mainly flow toward the central bed region after forming on the distributor surface. Therefore, the bubble flow is experienced in a lower extent by the particles moving along the wall (bubble-off position). The time-averaged voidage distribution can also support this. As seen in Figure 6, in the regions close to the walls, the time-averaged voidage corresponds to the jamming condition, which locally reduces particle movability. Consequently, one should expect lower velocity variance in this region. Another point discerned from Figure 22 is that the increase in the Bond number broadens the region with the low-velocity variance. This is associated with the voidage distribution plotted in Figure 6. As the Bond number increases, this “jamming” region expands toward the center and the distributor.

**Figure 23:**
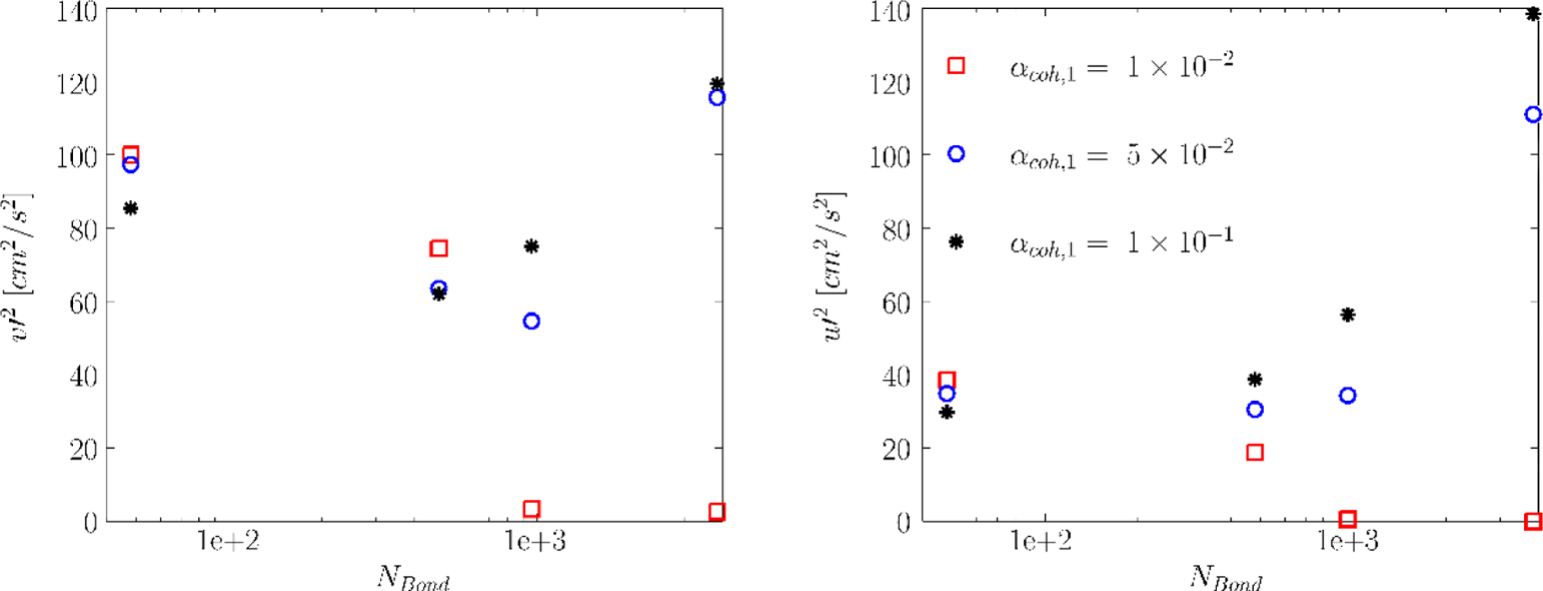
Effect of the Bond number and tensile stress parameter (α_coh,1_) on the time-averaged mean solids axial (left panel) and radial (right panel) velocity variances in the fluidized bed at *u* = 5*u*_mf_.

On the other hand, for α_coh,1_ = 1 × 10^–2^, a different trend was predicted. Specifically, an increase in the Bond number reduces the velocity variance in both axial and radial directions in such a way that for the cases bubble-less fluidization is experienced (e.g., *N*_Bo_ ≥ 960), the variances fall to zero. As discerned from Figure 23, the axial velocity variance decreases with the Bond number for *N*_Bo_ ≤ 960. However, higher velocity variance was predicted for *N*_Bo_ = 3840. Such a behavior can be supported by the instantaneous voidage distribution in the bed. As shown in Figure 12, relatively large bubbles are formed in the bed for this Bond number. Such large bubbles result in higher fluctuation in the particle axial velocity over time. Consequently, higher axial velocity variance can be expected. For a higher value of α_coh,1_, this behavior is observed at a lower Bond number of 960, as depicted in Figure 23. This means that the axial velocity variance hits a minimum at *N*_Bo_ = 480.

According to Figure 23, high Bond numbers (*N*_Bond_ ≥ 960) and high tensile pressure prefactors (α_coh,1_ ≥ 5 × 10^–2^) lead to a rise in the predicted *mean* velocity variances. This is to some degree counterintuitive as one would expect that these velocity fluctuations, and consequently the global mixing rate, reduce upon an increase in the particle level of cohesion. However, the mixing dynamics of wet granular matter are highly complex, as shown in many studies, e.g., Radl et al.^11^ The reason is that comparably large agglomerates when sheared at a given shear rate lead to a larger reference velocity simply because of dimensionality reasons. Also, kinetic theory of granular flow would predict increased granular temperature (and hence mixing) in the case of larger particles (or agglomerates) when considering a fixed shear rate.

The obtained trend of increased velocity variance at high Bond numbers can be explained to some degree by the contour plot of solids velocity variance in Figure 22 and time-averaged voidage distribution in Figure 6. As easily discerned in Figure 22, the axial solids velocity variance can be divided into two relatively uniform regions featuring (i) high-variance region in the central region of the bed and (ii) low-velocity variance developing from the wall to the central region of the fluidized bed. Considering the voidage contour plot in Figure 6, one can conclude that the high velocity variance is predicted in the region where voidage is relatively high due to the motion of bubbles. This can also be supported by the instantaneous voidage distribution in Figures 2 and 3. The reader is referred to Appendix C of the Supporting Information to see the motion of bubbles over time for a very cohesive bed. As we were unable to identify individual agglomerates in our present study, the exact mechanism that leads to increased velocity fluctuations at high Bond numbers could not be investigated in our present study.

## 4 Conclusions

The present study focused on the capability of the TFM approach in predicting the fluidization behavior of cohesive particles. To do so, the modified solids rheology for cohesive powders developed by Gu et al.^1^ was implemented in the MFiX platform. The main advantage of the implemented model of Gu et al.^1^ is that it is based on particle-level simulation of cohesive powder flow. This means that we do not need any empirical constitutive equation (e.g., agglomeration kernel in the population balance model) to predict particle agglomeration if the grid resolution is sufficiently fine. Specifically, this is since particle cohesion and agglomeration are already considered via tensile stress (i.e., a negative “tensile pressure”) in the solids rheology model of Gu.^1^

Our simulation results demonstrated that the TFM approach successfully predicted cohesive powder fluidization *for the first time*. The model was qualitatively validated against the data reported by Li et al.^11^ in terms of (i) the formation of particle clusters in the bubble and (ii) the formation of a solids tensile stress at the bubble cap. Most importantly, the flow of powders as clusters and agglomerates was predicted. Moreover, the distribution of negative pressure was presented, which can be attributed to the formation of tensile stress due to particle cohesion. Furthermore, our simulation results revealed that the particle Bond number and the tensile pressure prefactor critically affect the level of particle clustering in the fluidized bed. To thoroughly evaluate this, we performed a set of simulations for a wide range of particle cohesion levels to obtain a regime map of cohesive fluidization. Analyzing the voidage and pressure contour plot revealed that four different particle flow regimes are developed in the Bond number–tensile pressure prefactor space. The predicted flow regimes were successfully attributed to the competition between the acting shear stress and the yield stress of the powder that tends to form larger particle assemblies (i.e., agglomerates). Considering the cumulative distribution of τ_shear_/τ_yield_, the four observed regimes can be categorized as follows:

i Bubbling regime: the shear stress dominates the yield stress, resulting in no (or no significant) particle agglomeration.
ii Bubbling–clustering regime: the shear stress and the yield stress are of comparable magnitude such that the formation of particle clusters and agglomerates is expected mainly in bubbles.
iii Bubble-less expansion: the yield stress dominates the applied shear stress such that the fluidization gas cannot shear the particles. Hence, bubble formation is suppressed, and the bed becomes uniformly expanded.
iv Stationary bed: the yield stress is much larger than the applied shear stress such that the bed cannot get fluidized, and large agglomerates can be formed in the bed. Gas bypasses the particles through channeling. The computational cost for simulating such highly cohesive powders (e.g., high values of *N*_Bo_ or high values of α_coh,1_) is significantly larger than that for powders in the other regimes. Simulation can even become unstable due to the very small time step that is necessary to handle the extremely high negative pressures.

The solids rheology model developed by Gu et al.^1^ shows significant progress in the application of the TFM for cohesive fluidized beds. However, their model needs to be revisited for volume fractions beyond the jamming point. Their original model suggests high negative pressure at ε_s_ ≥ ε_c_, which is unphysical. In our present study, the pressure due to cohesion was set to zero for densely packed situations, that is, above the jamming point.

Apart from this, the analysis conducted in our present work should be extended in future studies. Most importantly, the regime map of fluidization for cohesive powders was obtained at two different fluidization velocities at constant particle and gas properties. However, this regime map should be extended to consider the effect of the dimensionless diameter (d*) and the dimensionless velocity (u*) similar to Grace’s fluidization chart.^26^ Furthermore, quantitative validation of the model requires (i) characterization of a set of powders with different cohesion levels to obtain accurate values of model parameters (e.g., the tensile and compression pressure prefactors) and subsequently (ii) a set of experiments using these powders in a fluidized bed.

Finally, predicting the agglomerate size would require a deeper analysis, which necessitates a detailed analysis of solids tensile pressure, as well as shear stress and yield stress. Specifically, the gradients of these quantities need to be tracked over time to evaluate if the particles have enduring contacts. The boundary between a specific agglomerate and the fluid could then be demarcated in a next step. One agglomerate can only be larger than one computational cell. Therefore, to compute the agglomerate size, one needs to perform an image analysis of solids volume fraction, solids tensile pressure, and yield stress to extract the agglomerate size. As the main focus of our present work is demonstrating the capability of the TFM approach in predicting agglomeration phenomena of a cohesive powder, the analysis of the agglomerate size distribution was postponed to a future study.

## Supporting Information Available

Original Gu’s model performance under highly closed pack conditions, distribution of voidage and tensile pressure representing solids flow path, particle agglomerate, and granule breakage limited by solids tensile pressure (PDF)
Distribution of voidage and tensile pressure (AVI)
Agglomerate breakup (AVI)

## Supplementary Material

ie1c04786_si_001.pdf

ie1c04786_si_002.avi

ie1c04786_si_003.avi

## Abbreviations

*C*_d_: drag coefficient
*d*_p_: diameter of the particles; m
*D*_gij_: rate of the strain tensor, fluid phase; s^–1^
*D*_sij_: rate of the strain tensor, solid phase; s^–1^
*D*_gn_: diffusion coefficient of *n*th gas-phase species; kg/m·s
*D*_wv_: molecular diffusivity of vapor in air; m^2^/s
*e*_eff_: effective coefficient of restitution for the collisions in solid phases
*e*_p,p_: coefficient of restitution for the collisions in solid phases
*e*_p,w_: coefficient of restitution for the collisions between solid particles and wall
*g*_i_: acceleration due to gravity; m/s^2^
*g*_0_: radial distribution function at the contact
*H*_0_: static bed height; m
*H*_bed_: bed height; m
*H*_exp_: expanded bed height; m
*i*,*j*: indices to identify vector and tensor components; summation convention is used only for these indices
*I*_gsi_: interphase momentum exchange force; N/m^3^
*J*_s_: granular energy transfer; m^2^/s^3^
*k*_g_: fluid-phase conductivity; J/m·K·s
*k*_s_: solid-phase conductivity; J/m·K·s
*L*_bed_: bed length; m
*n*: constant in the friction model
*N*_Bo_: Bond number; 6σ/ρ_p_*gd*_p_2
*P*_g_: pressure in the fluid phase; Pa
*P*_s,f_: frictional pressure in the solid phase; Pa
*P*_s_: solid pressure; Pa
Pr: Prandtl number; *C*_p_ν_f_ρ_f_/*k*_g_
Re_p_: solid-phase particle Reynolds number
Re_m_: mean particle Reynolds number
***S***: rate of deformation tensor; 1/s
*S*_gij_: gas-phase shear rate; s^–1^
*S*_sij_: solid-phase shear rate; s^–1^
*t*: time; s
*u*_mf_: minimum fluidization velocity; m/s
*U*_gi_: fluid-phase velocity vector; m/s
*u*_g_: superficial gas velocity; m/s
*U*_si_: solid-phase velocity vector; m/s
*W*(*x*): W-Lambert function
*W*_bed_: bed width; *m*

## Greek Letters

α: constant with a value of 1.6; dimensionless
γ_gm_: fluid–solid heat transfer coefficient corrected for interphase mass transfer; J/m^3^·K·s
β: mass transfer coefficient; m/s
β_gs_: coefficient for the interphase force between the fluid phase and the solid phase; kg/m^3^·s
δ_ij_: Kronecker delta function
Δ_Cell_: grid size; m
ε_g_: volume fraction of the fluid phase (void fraction)
ε_g_^*^: volume fraction of the fluid phase under minimum fluidization conditions
ε_s,max_: packed-bed (maximum) solid volume fraction
ε_s_: volume fraction of the solid phase
η: function of the restitution coefficient; (1 + *e*_pp_)/2
Θ: granular temperature of the solid phase; m^2^/s^2^
κ_s_: granular energy diffusion coefficient; kg/m·s
λ_rm_: solid conductivity function
μ_g_: molecular viscosity of the fluid phase; kg/m·s
μ_s_: solid-phase shear viscosity; kg/m·s
μ_slid_: sliding friction coefficient
μ_s,f_: frictional shear viscosity of the solid phase; kg/m·s
ξ: bulk viscosity; kg/m·s
Π_s_: exchange force in the granular energy equation; kg/m·s^3^
ρ_g_: microscopic (material) density of the fluid phase; kg/m^3^
ρ_s_: microscopic (material) density of the *m*th solid phase; kg/m^3^
τ_y_: yield stress; Pa
fluid-phase stress tensor; Pa
solid-phase stress tensor; Pa
solid-phase frictional stress tensor; Pa
Φ_p_: particle coating number
φ: angle of internal friction, also used as a general scalar
φ_s_: specularity coefficient
ψ_liq_: particle surface coverage

## Subscripts

exp: expanded bed
g: gas phase
init: at time of zero
max: maximum
mix: mixture
*p*: particle
*s*: solid phase

## superscripts

coh: cohesion force
max: maximum
norm: normalized

## Abbreviations

CFD: computational fluid dynamics
DEM: discrete element method
DNS: direct numerical simulation
EE: Euler–Euler (method)
EL: Euler–Lagrange (method)
FB: fluidized bed
KTGF: kinetic theory of granular flow
MFiX: Multiphase Flow with Interphase eXchange
