# Supplementary material for: Capability of the TFM Approach to Predict Fluidization of Cohesive Powders
Source: Ind Eng Chem Res. 2022 Feb 16;61(8):3186–205. doi: 10.1021/acs.iecr.1c04786 (PMC8895407; doi:10.1021/acs.iecr.1c04786)
Supplement: Supplementary file 1 — ie1c04786_si_001.pdf [file ie1c04786_si_001.pdf]

Supporting Information

Capability of the TFM approach to predict fluidization of cohesive powders

Maryam Askarishahi[1*], Mohammad-Sadegh Salehi[2], Stefan Radl[2]

[1]Research Center Pharmaceutical Engineering GmbH, Inffeldgasse 13/III, 8010 Graz, Austria

[2]Institute of Process and Particle Engineering, Graz University of Technology, Inffeldgasse 13/III, 8010 Graz, Austria

* Corresponding author: maryam.askarishahi@rcpe.at

Original Gu's model performance for highly closed pack conditions

This appendix deals with the pressure for highly closed pack conditions in the rheological model of Gu et al.[1] as explained in the manuscript, the pressure can diverge at $\varepsilon_s = \varepsilon_c$ and give unphysical negative pressure at $\varepsilon_s > \varepsilon_c$. Therefore, we limited the pressure due to cohesion to zero for $\varepsilon_s \geq \varepsilon_c$. This can prevent unphysical negative pressure for packed condition above jamming condition. To support this, a set of simulation was performed with the original format of pressure form Gu's model. The simulation results have been presented in Figure S. 1and Figure S. 2. As shown in this Figures, for Bond number in the range of $0.6 \leq N_{Bo} \leq 30$, particle agglomerates (indicated by small back regions in the bubble) can be observed in the bubbles with the lowest voidage and the most negative pressure. This means that such a negative pressure can suck out the particles so that the pressure can be balanced for a stable granule. This behavior does not seem realistic. Instead, we need to limit the pressure to zero for packed conditions above jamming condition.

We note in passing that such an unrealistic behavior increases the computational cost and even makes the simulation unstable at some points. However, the imposed limitation can also speed up the simulation.

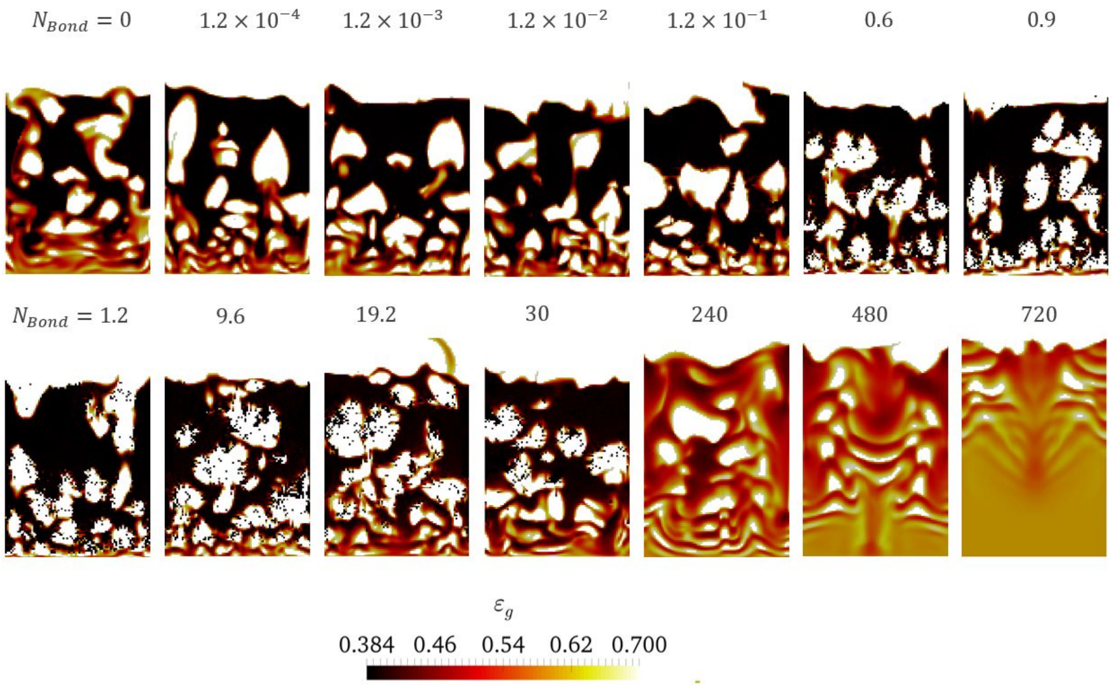

Figure S. 1-Voidage distribution for the fluidized bed with different Bond Numbers at fluidization velocity of 5u_mf for dry powders at time of 1.5s

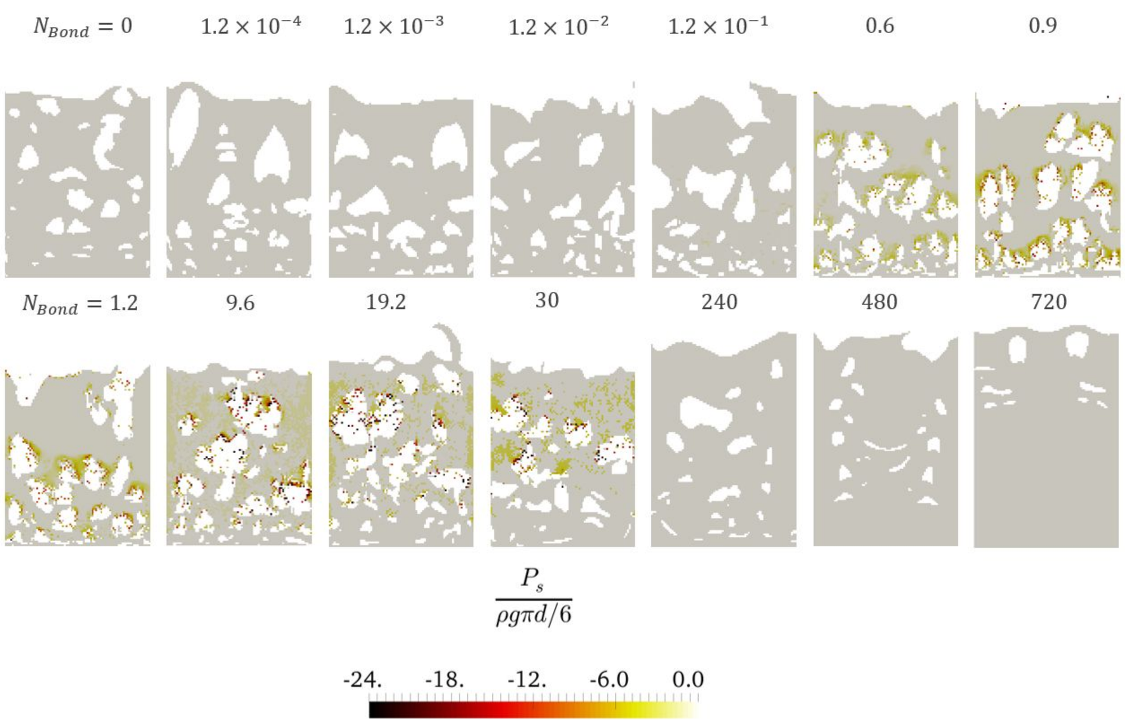

Figure S. 2-negative pressure distribution for the fluidized bed with different Bond Numbers at fluidization velocity of 5u_mf for dry powders at time of 1.5s
